# The *cssR* gene of *Corynebacterium glutamicum* plays a negative regulatory role in stress responses

**DOI:** 10.1186/s12934-021-01600-8

**Published:** 2021-06-03

**Authors:** Yang Liu, Wenzhi Yang, Tao Su, Chengchuan Che, Guizhi Li, Can Chen, Meiru Si

**Affiliations:** 1 College of Life Sciences, Qufu Normal University, Qufu, 273165 Shandong China; 2 Key Laboratory of Plant Genetics and Molecular Breeding, Henan Key Laboratory of Crop Molecular Breeding & Bioreactor, College of Life Science and Agronomy, Zhoukou Normal University, Zhoukou, 466001 Henan China; 3 School of Food Science and Nutrition, University of Leeds, Leeds, LS2 9JT UK

**Keywords:** Stress response, TetR, Transcription regulation, Ligand binding, *Corynebacterium glutamicum*

## Abstract

**Background:**

CssR, the product of the *Corynebacterium glutamicum ncgl1578* gene cotranscribed with *ncgl1579*, is a TetR (tetracycline regulator) family repressor. Although many TetR-type regulators in *C. glutamicum* have been extensively described, members of the TetR family involved in the stress response remain unidentified.

**Results:**

In this study, we found that CssR regulated the transcription of its own gene and the *ncgl1576-ncgl1577* operon. The *ncgl1576-ncgl1577* operon, which is located upstream of *cssR* in the orientation opposite that of the *cssR* operon, encodes an ATP-binding cassette (ABC), some of which are involved in the export of a wide range of antimicrobial compounds. The *cssR*-deletion (Δ*cssR*) mutant displayed increased resistance to various stresses. An imperfect palindromic motif (5′-TAA(G)TGN_13_CA(G)TTA-3′; 25 bp) located at the intergenic region between *cssR* and *ncgl1577* was identified as the sole binding site for CssR. Expression of *cssR* and *ncgl1577* was induced by antibiotics and heavy metals but not H_2_O_2_ or diamide, and the DNA-binding activity of CssR was impaired by antibiotics and heavy metals but not H_2_O_2_. Antibiotics and heavy metals caused CssR dissociation from target gene promoters, thus derepressing their transcription. Oxidant treatment neither altered the conformation of CssR nor modified its cysteine residues, indicating that the cysteine residues in CssR have no redox activity. In the Δ*cssR* mutant strain, genes involved in redox homeostasis also showed increased transcription levels, and the NADPH/NADP^+^ ratio was higher than that of the parental strain.

**Conclusion:**

The stress response mechanism of CssR in *C. glutamicum* is realized via ligand-induced conformational changes of the protein, not via cysteine oxidation-based thiol modification. Moreover, the crucial role of CssR in the stress response was demonstrated by negatively controlling the expression of the *ncgl1576-ncgl1577* operon, its structural gene, and/or redox homeostasis-related genes.

**Supplementary Information:**

The online version contains supplementary material available at 10.1186/s12934-021-01600-8.

## Background

*Corynebacterium glutamicum*, a well-known l-amino acid producer in industry and a model organism for systems biology, unavoidably generates or encounters a series of unfavorable circumstances during fermentation [1, 2], including pH and temperature fluctuations, osmotic variation, and nutrient shortages. Diverse environments inevitably produce excessive reactive oxygen species (ROS) [3]. Massive amounts of ROS are presumed to be toxic to cells such that they damage diverse cellular components, including DNA, lipids, proteins, iron sulfur clusters and the amino acids cysteine and methionine [4]. However, one of the most remarkable features of *C. glutamicum* is its striking survivability under excessive ROS production. As a result, the defense systems of *C. glutamicum* against stress-causing factors have attracted considerable attention from scientists, and their molecular mechanisms are now being revealed.

*Corynebacterium glutamicum* shows strong survivability that is attributed to two elaborate defense mechanisms, including enzymatic and nonenzymatic systems. In response to ROS, *C. glutamicum* mainly activates low-molecular-weight (LMW) substances as nonenzymatic systems, including β-carotene, vitamins (vitamins C and E), NAD(P)H, and LMW thiols, such as MSH (mycothiol; chemically, 1D-myo-inosityl-2-[*N*-acetyl-l-cysteinyl] amido-2-deoxy-α-d-glucopyranoside), cysteine, and coenzyme A [1, 5]. During the course of the defense response against ROS, the cellular concentration of NAD(P)H is critical because NAD(P)H serves as the main source of reducing power [6]. Millimolar concentrations of MSH constitute a buffer to maintain intracellular redox homeostasis, allow the proper functioning of a variety of biological molecules and prevent disulfide stress [1]. Along with the use of nonenzymatic systems, *C. glutamicum* leverages various direct ROS-scavenging terminal enzymes, oxidized protein-repairing oxidoreductases, and regulatory proteins. The enzymatic system for scavenging ROS involves a number of enzyme-catalyzed reactions with different mechanisms, such as superoxide dismutase (SOD), catalase (Kat) and peroxidases [7–11]. A series of peroxidases constitute a large family, including mycothiol peroxidase (MPx), peroxiredoxin (Prx), cysteine-based organic hydroperoxide resistance protein (Ohr), and osmotically inducible protein C (OsmC), which have been found to contribute to organism resistance to inorganic peroxide and organic hydroperoxide by detoxifying peroxides [8–11]. Peroxidases metabolize peroxides via a conserved NH_2_-terminal cysteine residue, which undergoes oxidation. To complete the catalytic cycle, the Cys residue must be reduced. Various peroxidases rely on different reducing systems, such as thioredoxin (Trx) and thioredoxin reductase (TrxR); mycoredoxin-1 (Mrx1), mycothione reductase (Mtr), mycothiol (MSH); alkyl hydroperoxide reductase subunit D (AhpD); dihydrolipoamide dehydrogenase (Lpd); and dihydrolipoamide succinyltransferase (SucB) [12–14]. Notably, when bacteria encounter stress, the expression levels of terminal peroxidases and oxidoreductases are altered, and this process is generally considered a stress response. To realize this response, the genes encoding these enzymes are regulated at the transcriptional level by stress response transcription factors. Each of these regulators senses a specific stress and responds to it by activating or derepressing a specific set of genes under its control. Frequently, the sensing of stress is mediated by oxidation of one or more regulator protein thiolates [15]. Certainly, regulator proteins also sense environmental stress by directly accommodating small ligands, such as salicylic acid (SA), antibiotics, and benzoate [16].

In *C. glutamicum*, many stress-sensing regulators from different transcription factor families, such as the LysR (DNA-binding transcriptional dual-lysine regulator) family regulator OxyR (the thiol-based redox sensor of peroxides) [17]; zinc-associated extracytoplasmic function (ECF)-type sigma factor H (SigH) [18]; MarR (multiple antibiotics resistance regulator) family of regulators, including RosR (regulator of oxidative stress response), OhsR (organic hydroperoxides stress regulator), CosR (*C. glutamicum* oxidant-sensing regulator), QorR (quinone oxidoreductase regulator), MalR (malic regulator), and OasR (organic peroxide- and antibiotic-sensing regulator) [19–24]; TetR (tetracycline repressor protein) family regulator OsrR (oxidative stress response regulator) [25]; and XRE (xenobiotic-response element) family regulator OsnR (oxidative stress negative regulator) [26], have been well studied. Among these regulatory protein families, the TetR family is one of the major transcription factor families in *C. glutamicum* [27]. In many cases, TetR family transcriptional regulators act as sensors to monitor the cellular environment in bacteria and provide a very common switch for the regulation of gene expression [28]. Many of these regulators control the expression of genes required for bacteria to adapt to environmental stresses [28]. However, research on TetR-type regulatory proteins in *C. glutamicum* is still very limited*.* Only several TetR-type regulators, including OsrR, the l-methionine biosynthesis repressor McbR, the resorcinol regulator RolR, the aconitase repressor AcnR, the *C. glutamicum* multidrug-responsive transcriptional repressor CgmR, the phenylacetic acid repressor PaaR, the biotin biosynthesis and transport repressor BioQ, and the ammonium assimilation and transport regulator AmtR, have been reported [25, 29–35]*.* Structural and functional analyses of these novel TetR family proteins can promote the elucidation of drug resistance mechanisms in bacteria. *C. glutamicum ncgl1578* encodes a protein that belongs to the helix-turn-helix DNA-binding motif-containing TetR family. NCgl1578, named CssR (*C. glutamicum* stress-sensing regulator) on the basis of the results of this study, is located immediately downstream and is oriented in the direction opposite that of the *ncgl1576-ncgl1577* operon, encoding the ATP-binding cassette (ABC). Importantly, CssR contains two cysteine residues. This characteristic allowed us to investigate the function of *C. glutamicum* CssR as a transcriptional repressor of putative toxic compound transporters critical for increasing resistance to environmental stresses. In the present study, CssR serving as a transcriptional repressor was found to directly control the expression of the *cssR-ncgl1579* and *ncgl1576-ncgl1577* operons and to indirect negatively control the genes involved in redox homeostasis. In addition, we showed that the responses of the *cssR-ncgl1579* and *ncgl1576-ncgl1577* operons were affected by antibiotics and heavy metals but not hydrogen peroxide (H_2_O_2_) or diamide. To our knowledge, this is the first report demonstrating the ability of CssR to sense intracellular stress.

## Methods

### Strains and culture conditions

The bacterial strains and plasmids used in this study were listed in Additional file 1: Table S1. *Escherichia coli* and *C. glutamicum* RES167 were cultured in Luria–Bertani (LB) medium as previously reported [36]. For generating and maintaining *C. glutamicum* RES167 mutants, brain heart infusion containing 0.5 M sorbitol (BHIS) medium was used [36]. Δ*cssR* and Δ*ncgl1576-ncgl1577* in-frame deletion mutants were generated by means of the method described [36]. For complementation, the pXMJ19-*cssR* and pXMJ19-*ncgl1576-ncgl1577* derivatives were transformed into Δ*cssR* and Δ*ncgl1576-ncgl1577* mutants by electroporation, respectively. The transformants were selected on LB plates supplemented with nalidixic acid (NAL) and chloramphenicol (CHL) and the expression was induced by adding 0.5 mM isopropyl β-d-1-thiogalactopyranoside (IPTG) into medium. All chemicals were of Analytical Reagent Grade purity or higher. Antibiotics were added at the following concentrations: Kanamycin (KAN), 50 µg ml^−1^ for *E. coli* and 25 µg ml^−1^ for *C. glutamicum*; NAL, 40 µg ml^−1^ for *C. glutamicum*; CHL, 20 µg ml^−1^ for *E. coli* and 10 µg ml^−1^ for *C. glutamicum*.

### Plasmid construction

The *cssR* and *ncgl1576*-*ncgl1577* genes were amplified by PCR from genomic DNA of *C. glutamicum* RES167 strain with corresponding primer pairs listed in Additional file 1: Table S2. These DNA fragments were digested and subcloned into similar digested vectors, obtaining pET28a-*cssR*, pXMJ19-*cssR* and pXMJ19-*ncgl1576*-*ncgl1577*.

The suicide plasmids pK18*mobsacB-*Δ*cssR* and pK18*mobsacB*-Δ*ncgl1576*-*ncgl1577* were prepared by overlap PCR with primer pairs listed in Additional file 1: Table S2 according to the method described by Shen et al. [36].

Site-directed mutagenesis was constructed as described [21].

The *lacZY* fusion reporter vectors pK18*mobsacB-P*_*cssR*_*::lacZY*, pK18*mobsacB-P*_*ncgl1577*_*::lacZY*, pK18*mobsacB-P*_*mshC*_*::lacZY*, and pK18*mobsacB-P*_*sodA*_*::lacZY* were made by the fusion of the promoter DNA fragments of *cssR* (152-bp, from − 140 to 12 bp), *ncgl1577* (155-bp, from − 143 to 12 bp), *mshC* (305-bp, from − 293 to 12 bp), and *sodA* (612-bp, from − 597 to 15 bp) [all distances were with respect to the start codon of the open reading frame (ORF) of the target gene] to the *lacZY* reporter gene via overlap PCR [21].

For obtaining pK18*mobsacB-P*_*cssRM*_*::lacZY*, 152-bp *cssR* promoter DNA containing mutagenesis sequence of the identified CssR binding site (*P*_*cssRM*_) was first directly synthesized by Shanghai Biotechnology Co., Ltd.. Mutagenesis sequence was shown in blue below the promoter sequence (Additional file 1: Figure S2a). *P*_*cssRM*_ had the same nucleotide sequence as 152-bp *P*_*cssR*_ in *P*_*cssR*_*::lacZY* except for mutation sites. Then, the resulting 152-bp *P*_*cssRM*_ was fused to a *lacZY* reporter gene*.* Finally, *P*_*cssRM*_*::lacZY* was inserted into similar digested pK18*mobsacB.* A similar process was used to construct pK18*mobsacB-P*_*ncgl1577M*_*::lacZY*.

The fidelity of all constructs was confirmed by DNA sequencing (Sangon Biotech, Shanghai, China).

### Overexpression and purification of recombinant protein

To express and purify soluble His_6_-tagged recombinant proteins, pET28a derivatives were transformed into BL21(DE3) cells. Recombinant proteins were purified with the His·Bind Ni–NTA resin (Novagen, Madison, WI) according to manufacturer’s instructions. Eluted recombinant proteins were dialyzed against PBS at 4 °C and concentrated for further experiments [> 95% purity as estimated by sodium dodecyl sulphate-polyacrylamide gel eletrophoresis (SDS-PAGE)]. Cleavage of the His_6_ tag was performed by adding 10 units of Enterokinase-Max (Invitrogen, Karlruhe, Germany) and incubation at 4 °C overnight to conduct subsequent isothermal titration calorimetry (ITC) analysis. Ni–NTA agarose was used to remove the cleaved tag and uncleaved protein from the tag-free protein. All enzymes were purchased from Sigma-Aldrich (St. Louis, MO).

### Sensitivity assays

For measuring the response to antibiotic, heavy metal and oxidant, experiment was performed according to Helbig et al. [37].

To measure the response to various environmental stress conditions, overnight cultures of *C. glutamicum* strains grown in LB broth medium at 30 °C were diluted 100-fold with LB broth medium, and the diluted cells were exposed to various antibiotics (1 μg ml^−1^ GEN, 3.5 μg ml^−1^ ERY, 1.2 μg ml^−1^ CIP for 60 min), oxidants (100 mM H_2_O_2_, 25 mM diamide, 5.5 mM CHP, and 15 mM *t*-BHP for 30 min) and heavy metals (0.15 mM CdCl_2_, 6 mM NiSO_4_, 0.5 mM K_2_Cr_2_O_7_ for 30 min) at 30 °C with shaking (100 rpm). After treatment, the cultures were serially diluted and plated onto LB agar plates, and colonies were counted after 36 h of growth at 30 °C. Percentage survival was calculated as follows: [(CFU (Colony-Forming Unit) ml^−1^ after challenge at different stresses)/(CFU ml^−1^ before stress challenge)] × 100.

### Ligand binding assays

Ligand binding was measured using isothermal titration calorimetry (ITC) at 25 °C with a NANO-ITC 2G microcalorimeter (TA Instruments, New Castle, DE, USA) [24].

### Construction of chromosomal fusion reporter strains and β-galactosidase assay

The *lacZY* fusion reporter plasmids pK18*mobsacB*-*P*_*cssR*_*::lacZY*, pK18*mobsacB*-*P*_*ncgl1577*_*::lacZY*, pK18*mobsacB*-*P*_*cssRM*_*::lacZY*, and pK18*mobsacB*-*P*_*ncgl1577M*_*::lacZY* were transformed into corresponding *C. glutamicum* RES167 strains by electroporation. The transformants were selected by plating on LB agar plates containing 40 µg ml^−1^ NAL, 25 µg ml^−1^ KAN, and 10 µg ml^−1^ CHL [24]. The resulted strains were grown in LB broth medium to an optical density at 600 nm of 0.6–0.7 and then treated with different reagents of various concentrations at 30 °C for 30 min. β-Galactosidase activities were assayed with *O*-nitrophenyl-β-d-galactopyranoside (ONPG) as the substrate [38]. All β-galactosidase experiments were performed with at least three independent biological replicates.

### Quantitative real-time polymerase chain reaction (qRT-PCR) analysis

Total RNA was isolated from exponentially growing strains exposed to different toxic agents of indicated concentrations for 30 min using the RNeasy Mini Kit (Qiagen, Hilden, Germany) along with the DNase I Kit (Sigma-Aldrich, Taufkirchen, Germany). Purified RNA was reverse-transcribed with random 9-mer primers and MLV reverse transcriptase (TaKaRa, Dalian, China). Quantitative RT-PCR analysis (7500 Fast Real-Time PCR; Applied Biosystems, Foster City, CA) was performed as described previously [20]. The primers used were listed in Additional file 1: Table S2. To obtain standardization of results, the relative abundance of 16S rRNA was used as the internal standard. The experiment was performed with at least three independent biological replicates.

### Electrophoretic mobility shift assay (EMSA)

EMSA was performed using the method of Si et al. [24]. Briefly, 131-bp *cssR* DNA promoter sequence [*P*_*cssR*_; covering the putative promoter sequences of the *cssR-ncgl1579* and *ncgl1576-ncgl1577* operons and corresponding to nucleotides − 131 to − 1 relative to the translational start codon (ATG) of the *cssR* ORF] was amplified using primer pair EcssR-F/EcssR-R (Additional file 1: Table S2). A 131-bp mutation promoter DNA sequence (*P*_*cssRM*_) was directly synthesized by Shanghai Biotechnology Co., Ltd. *P*_*cssRM*_ contained the mutated sequence of the identified CssR-binding site (the mutated sequence was shown in blue below the promoter sequence in Additional file 1: Figure S2a) and had the same nucleotide sequence as 131-bp *P*_*cssR*_ except for mutation sites. The binding reaction mixture (20 μl) contained 10 mM Tris–HCl (pH 7.4), 5 mM MgCl_2_, 50 mM KCl, 5% glycerol, 0.1% Nonidet P 40 (NP40), 1 μg poly(dI:dC), 0.5 mM ethylene diamine tetraacetic acid (EDTA), 20 ng *P*_*cssR*_ or *P*_*cssRM*_, and 0–80 nM of CssR. After the binding reaction mixture was incubated at room temperature for 30 min, the mixture was subjected to electrophoresis on 8% nondenaturing polyacrylamide gel, and stained with a 10,000-fold diluted SYBR Gold nucleic acid staining solution (Molecular Probes) for 30 min. The DNA bands were visualized with UV light at 254 nm. Fragments amplified from the *cssR* coding region with primers Control-F and Control-R instead of the 131-bp *cssR* promoter or BSA instead of His_6_-CssR in the binding assays were used as negative controls to determine the binding specificity of CssR.

The loss of binding due to xenobiotics inducer was tested as follows. Indicated concentration of xenobiotics were added to CssR solution, immediately aliquots were taken and was cultivated with 20 ng *P*_*cssR*_ for EMSA. All aliquots were incubated in binding buffer for 30 min at room temperature and separated on 8% nondenaturing polyacrylamide gel and the gel was stained using SYBR Gold nucleic acid staining solution. The experiment was performed in triplicate.

For the determination of apparent *K*_*D*_ values, increasing concentrations of the CssR (0–100 nM) were incubated for 30 min at room temperature with 20 ng *P*_*cssR*_. The samples were applied onto an 8% native polyacrylamide gel and separated at 180 V for 1 h on ice. The gels stained with GelRed™ and photographed were quantified using ImageQuant software (GE Healthcare), and the percentage of shifted DNA was calculated. These values were plotted against the CssR concentration in log_10_ scale, and a sigmoidal fit was performed using GraphPad Prism software (GraphPad Software, San Diego California USA), considering the error bars as well as 0 and 100% shifted DNA as asymptotes, the turning point of the curve was defined as the apparent *K*_*D*_ value. All determinations were performed in triplicate.

### DNase I footprinting assay

DNase I footprinting assays were performed as described [39].

### Size exclusion chromatography

The size of purified His_6_-CssR was estimated by gel filtration on Superdex 75 10/300 GL column (GE Healthcare, Piscataway, NJ) using a buffer (50 mM potassium phosphate [pH 7.4], 0.15 M NaCl) with a gel filtration calibration kit (low molecular weight; GE, United Kingdom). The calibration curve was plotted by use of the *K*_av_ versus the logarithm of the molecular weight.

### Quantification of intracellular NADPH/NADP^+^ and NADH/NAD^+^ ratios

NADPH/NADP^+^ and NADH/NAD^+^ ratios were detected according to the cold methanol quenching method described by Jeong et al. using the NADPH/NADP^+^ and NADH/NAD^+^ assay kit (Bioassay systems, USA) [26]. Intracellular nucleotides were extracted according to the manufacturer’s protocol. The assays utilized alcohol dehydrogenase and glucose dehydrogenase cycling reactions for NADP(H) and NAD(H) quantification, respectively. Colorimetric changes were measured at 565 nm using a Shimadzu UV-1650 PC spectrophotometer. The experiment was performed with at least three independent biological replicates.

### Western blot analysis

Western blot analysis was performed as described previously [20]. The primary antibody at 4 °C overnight: anti-NCgl1577 rabbit polyclonal antibody, 1:1000; anti-NCgl1579 rabbit polyclonal antibody, 1:1000; anti-cytosolic RNA polymerase α (α-RNAP), 1:5000. The α-RNAP was used as a loading control. The anti-NCgl1577 and anti-NCgl1579 rabbit polyclonal antibodies were generated and affinity-purified according to the method described previously [40]. The density of bands on Western blots was quantified by Image Lab (Bio-Rad, California, USA).

### Quantitative analysis of sulfhydryl groups

Free thiol content of CssR was measured by using 5,5′-dithio-bis (2-nitrobenzoic acid) (DTNB) [41, 42].

### The redox state of CssR

The redox state of CssR (20 μM) was analyzed by incubating the proteins with 50 mM DTT, 100 μM H_2_O_2_, 80 μM CHP, and 60 μM diamide for 30 min before separating on nonreducing 15% SDS-PAGE. For nonreducing conditions, the loading buffer [250 mM Tris–HCl (pH 6.8), 0.5% bromophenol blue (BPB), and 50% glycerol] was added to treated protein samples. All the samples were boiled for 5 min prior to electrophoresis and then stained with Coomassie Brilliant Blue (CBB). The experiment was performed in triplicate.

### Statistical analysis

Statistical analyses of survival rate, transcription level and protein level were determined with paired two-tailed Student’s t-test. GraphPad Prism Software was used to carry out statistical analyses (GraphPad Software, San Diego California USA).

## Results

### The TetR-type regulator CssR was conserved in corynebacteria

It has been reported that TetR family regulators function as negative regulators of physiological processes such as efflux pumping and biosynthesis of antibiotics, osmotic stress, and solvent resistance [28]. The *ncgl1578* gene of *C. glutamicum*, which was renamed *cssR* (*C. glutamicum* stress-sensing regulator) on the basis of the observed phenotypes described herein, encodes a putative TetR-type transcriptional regulator of 198 amino acids (mass, 21,786 Da). A Pfam analysis showed that the deduced CssR protein possessed a TetR-type helix-turn-helix motif located near the N-terminal region (amino acid residues 12 to 50). A sequence comparison showed that CssR putative homologs were present in several species of the genus *Corynebacterium*, such as *C. deserti*, *C. crudilactis*, *C. callunae*, and *C. halotolerans* (Additional file 1: Figure S1a). Notably, the genomic organization of the *cssR* gene in *C. glutamicum* was almost identical to that of *C. deserti*, *C. crudilactis*, *C. callunae*, and *C. halotolerans* (Additional file 1: Figure S1b).

### Involvement of *cssR* in antibiotic, heavy metal, and oxidant stress responses

In the genome of *C. glutamicum*, *cssR* was organized in an operon with *ncgl1579*, which encodes a putative protein. An amino acid sequence comparison showed that NCgl1579 has amino acid identities of more than 45% with cystathionine beta-synthase (CBS) domain-containing proteins in *C. suranareeae*, *C. glaucum*, and *C. uterequi* (Additional file 1: Figure S1c). CBS is a key enzyme in the metabolic pathway of homocysteine *trans*-sulfurization [43]. This finding suggested that NCgl1579 was involved in stress resistance by affecting cysteine synthesis. Ninety-three base pairs upstream of *cssR* constitute the *ncgl1576*-*ncgl1577* locus, which is oriented in the direction opposite that of *cssR* (Additional file 1: Figure S2a). The *ncgl1576*-*ncgl1577* operon encodes a putative ATP-binding cassette (ABC) transporter permease (NCgl1576) and a putative ABC transporter ATP-binding protein (NCgl1577). Many ABC transport proteins have been found to be involved in the export of a wide range of antimicrobial compounds and have been implicated in the stress response [44]. In several cases, bacterial drug transporter proteins have been described as being controlled by a transcriptional regulatory protein often located in the same operon or in an immediately adjacent region and in the orientation opposite that of the target gene on the chromosome [45]. This genetic organization allowed us to speculate that CssR might also be involved in the stress response.

**Fig. 1:**
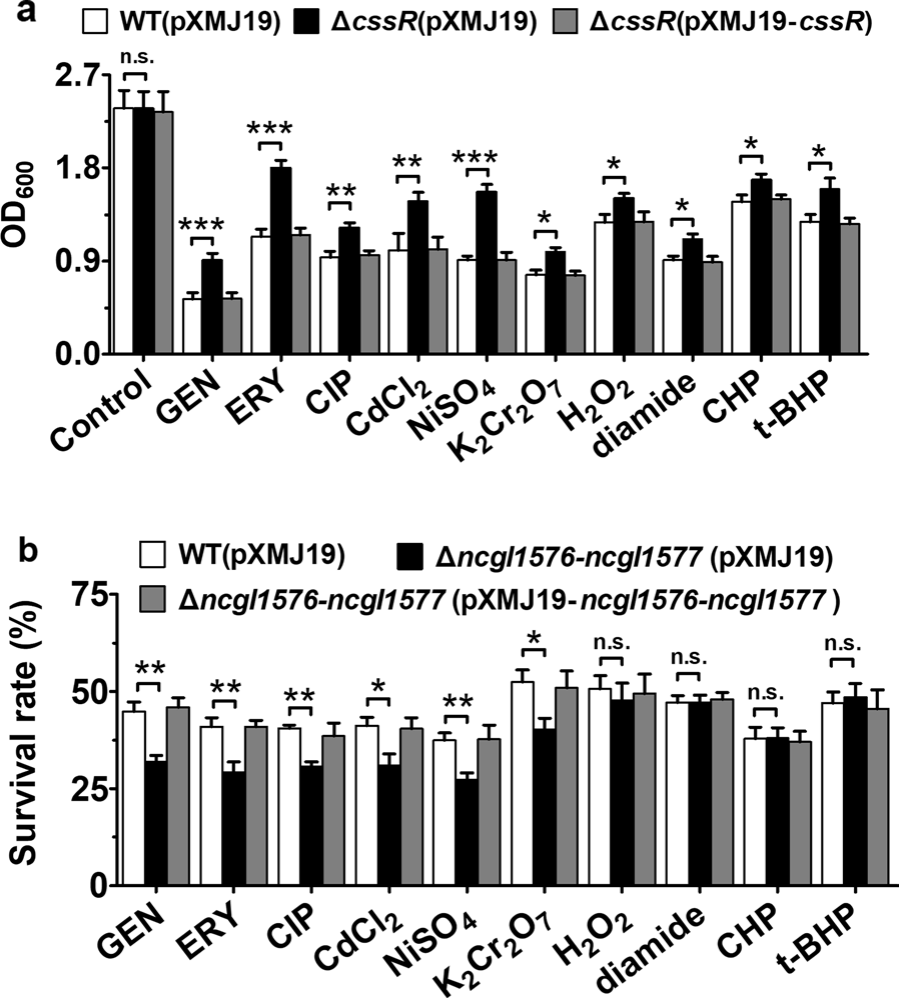
CssR was involved in stress resistance. **a** The growth (OD_600_) of the WT(pXMJ19) (*C. glutamicum* RES167 parental strain with the empty plasmid pXMJ19), Δ*cssR*(pXMJ19) (the mutant lacking *cssR* with the empty plasmid pXMJ19), and Δ*cssR*(pXMJ19-*cssR*) (the Δ*cssR* mutant expressed the wild-type *cssR* gene with a shuttle vector pXMJ19) strains after 24 h at 30 °C in LB broth medium containing 0.3 μg ml^−1^ gentamicin (GEN), 1.1 μg ml^−1^ erythromycin (ERY), 0.4 μg ml^−1^ ciprofloxacin (CIP), 40 μM cadmium chloride (CdCl_2_), 2 mM nickel sulfate (NiSO_4_), 0.1 mM potassium dichromate (K_2_Cr_2_O_7_), 50 mM hydrogen peroxide (H_2_O_2_), 5 mM diamide, 0.3 mM cumene hydroperoxide (CHP), and 1.5 mM *tet*-butyl hydroperoxide (*t*-BHP), respectively, was recorded. The growth in LB broth medium without agents was used as control. **b** The WT(pXMJ19), Δ*ncgl1576-ncgl1577*(pXMJ19) (the mutant lacking *ncgl1576*-*ncgl1577* with the empty plasmid pXMJ19), and Δ*ncgl1576-ncgl1577*(pXMJ19-*ncgl1576-ncgl1577*) (the Δ*ncgl1576-ncgl1577* mutant expressed the wild-type *ncgl1576*-*ncgl1577* gene with a shuttle vector pXMJ19) strains grown to the stationary phase were exposed to indicated agents for 60 or 30 min at 30 °C, respectively. The viability of the cells was determined. Data shown were the averages of three independent experiments, and error bars indicated the SDs from three independent experiments. *P < 0.05; **P < 0.01; ***P < 0.001. *n.s.* no significance

To elucidate its role in physiology, we constructed *C. glutamicum cssR* deletion and complement strains through gene disruption and complementation and then analyzed the survival rate of the mutant strains under various stresses (Additional file 1: Figure S2b). Although *the C. glutamicum* RES167 parental strain (WT) and Δ*cssR* mutant showed almost identical growth rates (Additional file 1: Figure S3), the sensitivity of the Δ*cssR*(pXMJ19) mutant (the mutant lacking the *cssR* gene and expressing the empty plasmid pXMJ19) to various agents was remarkably lower than that of WT(pXMJ19) (the *C. glutamicum* RES167 parental strain expressing the empty plasmid pXMJ19) and Δ*cssR*(pXMJ19-*cssR*) (the Δ*cssR* mutant expressing the wild-type *cssR* gene and the shuttle vector pXMJ19) (Fig. 1a). These results showed that deletion of the *cssR* gene led to increased resistance to agents such as antibiotics, heavy metals, and oxidants, further indicating that CssR functioned to combat cellular stress induced by a wide spectrum of environmental cues and played a negative regulatory role in stress response-related genes.

Next, we constructed *C. glutamicum ncgl1576* and *ncgl1577* deletion and the complement strain and then analyzed their survival rates under various stress conditions (Additional file 1: Figure S2b). Although the Δ*ncgl1576*-*ncgl1577* mutant did not exhibit changes in growth under normal conditions (Additional file 1: Figure S3), mutants lacking NCgl1576 and NCgl1577 showed obvious sensitivity to gentamicin (GEN), erythromycin (ERY), ciprofloxacin (CIP), cadmium chloride (CdCl_2_), nickel sulfate (NiSO_4_), and potassium dichromate (K_2_Cr_2_O_7_) (Fig. 1b). Moreover, the complementary strains Δ*ncgl1576*-*ncgl1577* (pXMJ19-*ncgl1576*-*ncgl1577*) (the Δ*ncgl1576*-*ncgl1577* mutant expressing the wild-type *ncgl1576*-*ncgl1577* gene and a shuttle vector pXMJ19) showed a survival rate similar to that of the WT (pXMJ19) strain. We also tested the effect of hydrogen peroxide (H_2_O_2_), diamide, cumene hydroperoxide (CHP), and *tert*-butyl hydroperoxide (*t*-BHP), but in these cases, we observed no significant differences between the tested strains (Fig. 1b). These results indicated the crucial function of the *ncgl1576*-*ncgl1577* operon for bacteria survival under antibiotic and heavy metal conditions.

### CssR negatively regulated the expression of the divergently oriented operons *ncgl1576-ncgl1577* and *cssR-ncgl1579*

**Fig. 2:**
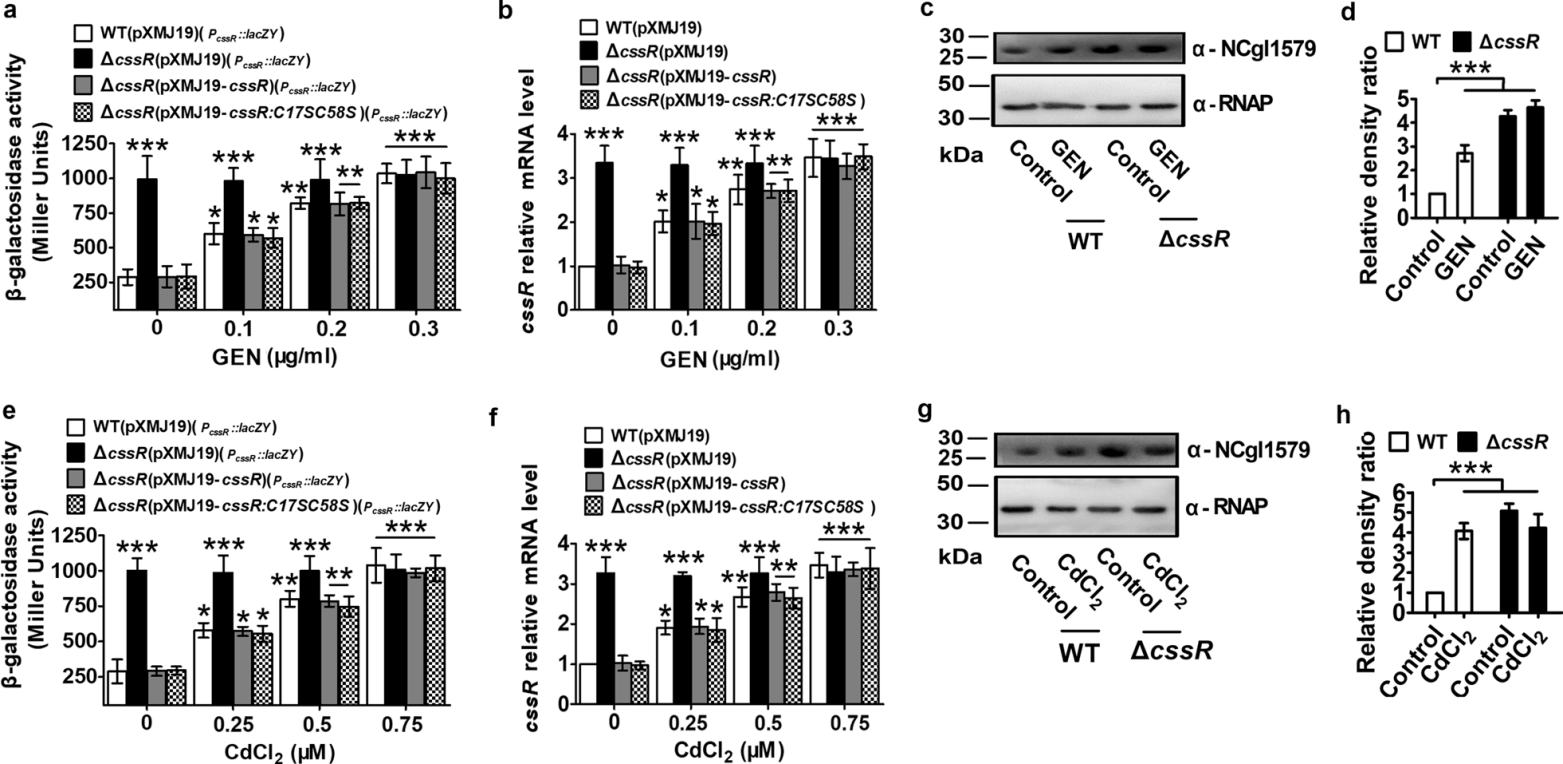
CssR was negatively autoregulated. **a**, **e** β-Galactosidase analyses of the *cssR* promoter activity by using the transcriptional *P*_*cssR*_*::lacZY* chromosomal fusion reporter expressed in WT(pXMJ19), Δ*cssR*(pXMJ19), and Δ*cssR*(pXMJ19-*cssR*) strains in the presence of gentamicin (GEN) or CdCl_2_ (cadmium chloride) conditions. **b**, **f** Quantitative RT-PCR analyses of *cssR* expression in WT(pXMJ19), Δ*cssR*(pXMJ19), and Δ*cssR*(pXMJ19-*cssR*) strains under GEN or CdCl_2_ conditions. The mRNA levels were presented relative to the value obtained from WT(pXMJ19) cells without stress treatment. Relative transcript levels of WT(pXMJ19) strains without stress treatment were set at a value of 1.0. **c**, **g** The protein levels of NCgl1579 in WT and Δ*cssR* in the presence or absence of GEN and CdCl_2_. Lysates from stationary phase bacteria exposed to GEN or CdCl_2_ for 2 h were resolved by SDS-PAGE, and NCgl1579 was detected by immunoblotting using specific anti-NCgl1579 antibody. For the pellet fraction, RNA polymerase α (α-RNAP) was used as a loading control. Similar results were obtained in three independent experiments, and data shown were from one representative experiment done in triplicate. **d**, **h** Relative quantified data for protein levels by Image Lab. Quantified protein expression of Western blots in **c** and **g**. Densities of proteins were all justified with α-RNAP. Relative density ratios of *C. glutamicum* RES167 parental strains (WT) without stress were set at a value of 1.0. Data shown were the averages of three independent experiments, and error bars indicated the SDs from three independent experiments. *P < 0.05; **P < 0.01; ***P < 0.001

**Fig. 3:**
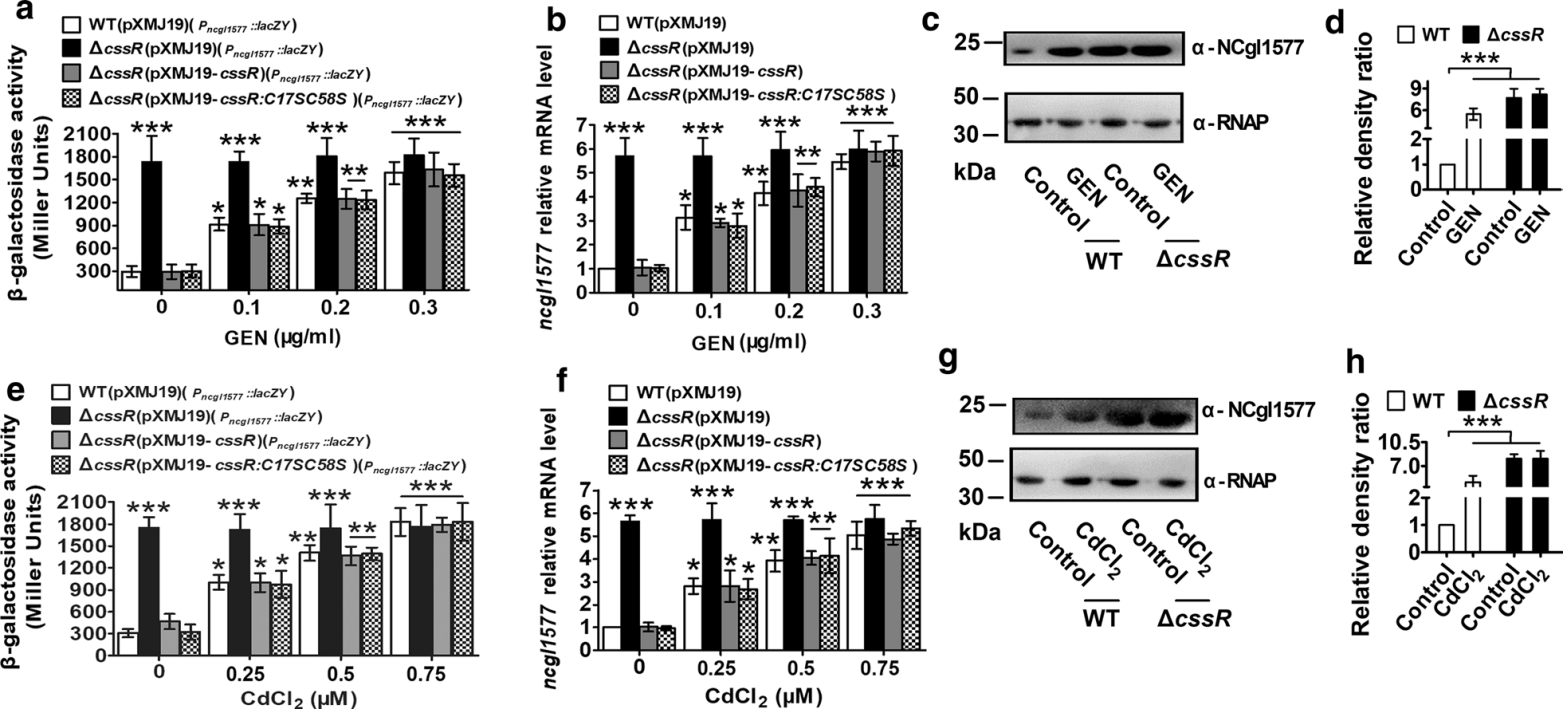
Negative regulation of *ncgl1577* by CssR. **a**, **e** β-galactosidase analyses of the *ncgl1577* promoter activities by using the transcriptional *P*_*ncgl1577*_*::lacZY* chromosomal fusion reporter expressed in WT(pXMJ19), Δ*cssR*(pXMJ19), and Δ*cssR*(pXMJ19-*cssR*) strains in the presence of GEN or CdCl_2_ conditions. **b**, **f** Quantitative RT-PCR analyses of *ncgl1577* expression in WT(pXMJ19), Δ*cssR*(pXMJ19), and Δ*cssR*(pXMJ19-*cssR*) strains under GEN or CdCl_2_ conditions. The mRNA levels were presented relative to the value obtained from WT(pXMJ19) cells without stress treatment. Relative transcript levels of WT(pXMJ19) strains without stress treatment were set at a value of 1.0. **c**, **g** The protein levels of NCgl1577 in WT and Δ*cssR* in the presence or absence of GEN or CdCl_2_. Lysates from stationary phase bacteria exposed to GEN or CdCl_2_ for 2 h were resolved by SDS-PAGE, and NCgl1577 was detected by immunoblotting using specific anti-NCgl1577 antibody. For the pellet fraction, α-RNAP was used as a loading control. Similar results were obtained in three independent experiments, and data shown were from one representative experiment done in triplicate. **d**, **h** Relative quantified data for protein levels by Image Lab. Quantified protein expression of western blots in **c** and **g**. Densities of proteins were all justified with α-RNAP. Relative density ratios of WT without stress were set at a value of 1.0. Data shown were the averages of three independent experiments, and error bars indicated the SDs from three independent experiments. *P < 0.05; **P < 0.01; ***P < 0.001

By the online software Virtual Footprint and PROM-Prediction of bacterial promoters, two putative overlapping and divergent promoter sequences in the intergenic region between the start codons of *cssR* and *ncgl1577* were found (Additional file 1: Figure S2a), one of which was located upstream of *cssR*. Neighboring *ncgl1577* had a putative − 10 and − 35 promoter sequence, which was found to be the *ncgl1577* promoter. Moreover, a putative CssR-binding site in the putative overlapping, divergent promoters of the *cssR*-*ncgl1579* and *ncgl1576-ncgl1577* operons was found (Additional file 1: Figure S2a). Thus, we speculated that CssR negatively regulated the *cssR*-*ncgl1579* operon and repressed the transcription of the adjacent, oppositely oriented *ncgl1576-ncgl1577* operon. To verify this speculation, *cssR* and *ncgl1577* transcription levels in the WT (pXMJ19), Δ*cssR* (pXMJ19), and Δ*cssR* (pXMJ19-*cssR*) strains were analyzed by qRT-PCR, and the *lac*Z*Y* activity of the chromosomal promoter fusion reporter was determined. Notably, to study the expression of *cssR* in the Δ*cssR*(pXMJ19) mutant by qRT-PCR, a 91-bp *cssR* transcript (corresponding to nucleotides + 1 to + 91 relative to the translational start codon (ATG) of the *cssR* gene) was amplified from the *cssR* ORF that remained in the Δ*cssR*(pXMJ19) mutant strain with the primers QcssR-F and QcssR-R (Additional file 1: Figure S4). As expected, the *cssR* and *ncgl1577* transcription levels in the Δ*cssR*(pXMJ19) mutant strain were obviously higher than those in the WT(pXMJ19) and Δ*cssR*(pXMJ19-*cssR*) strains (Figs. 2a, b, e, f, 3a, b, e, f). These results indicated that CssR negatively controlled the expression of the *ncgl1576*-*ncgl1577* operon and its structural gene.

### CssR regulated the expression of the *cssR-ncgl1579* and *ncgl1576-ncgl1577* operons by directly binding to their promoters

**Fig. 4:**
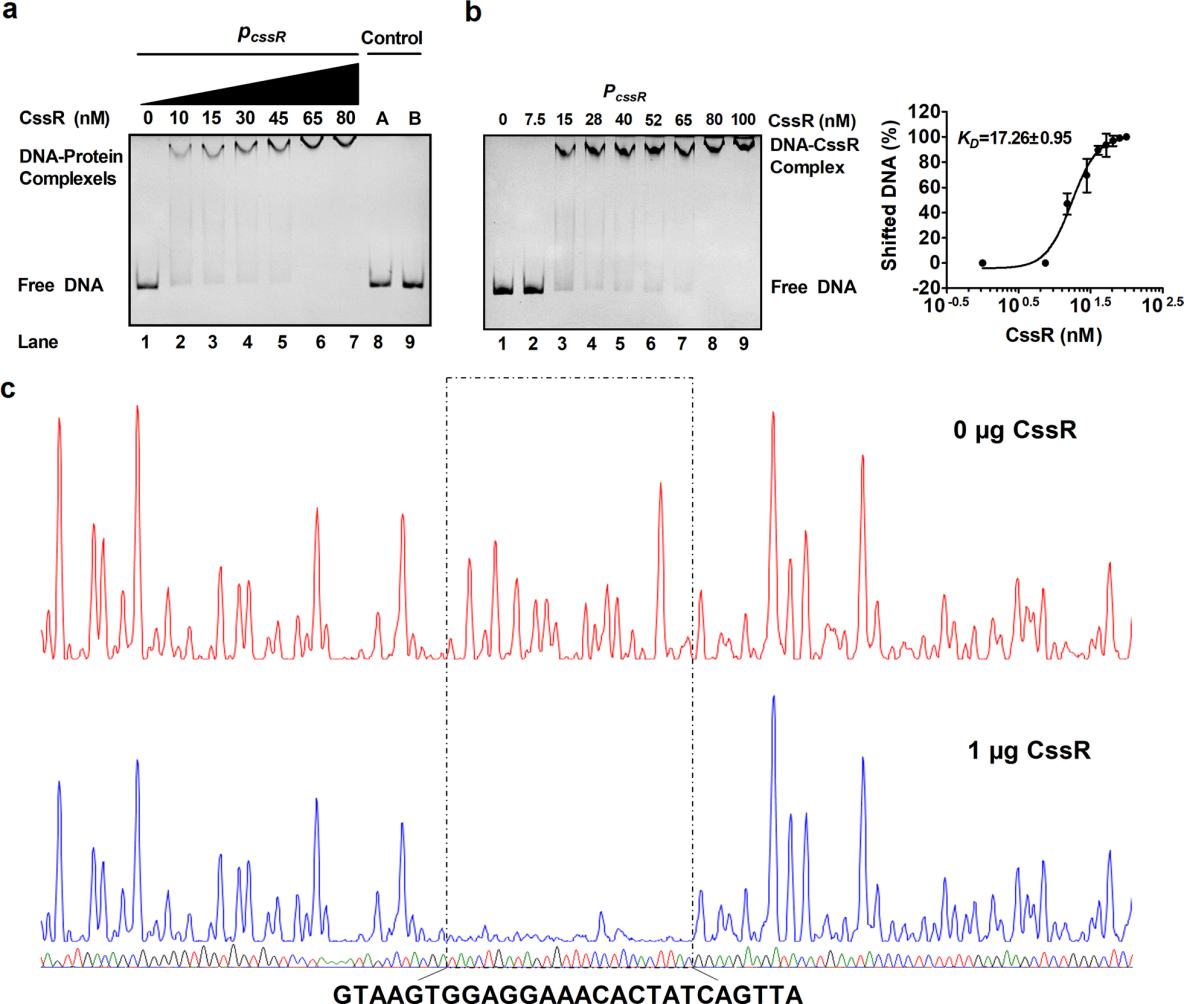
CssR bound directly to the promoter regions of the *cssR-ncgl1579* and *ncgl1576*-*ncgl1577* operons. **a** The interaction between His_6_-CssR and the 131-bp promoter fragment in the intergenic region between *cssR* and *ncgl1577* (named *P*_*cssR*_). A 131-bp fragment amplified from the *cssR* coding region using the primers control F and control R instead of the 131-bp *cssR* promoter (control A, lane 8) and an irrelevant protein BSA instead of CssR (control B, lane 9) in the binding assays were used as negative controls. **b** Determination of the apparent *K*_*D*_ value of CssR for 131-bp *P*_*cssR*_. 131-bp *P*_*cssR*_ was incubated with increasing CssR concentrations. At least three independent gels were performed for each binding site. The bands were quantified using ImageQuant software (GE Healthcare), and the percentage of shifted DNA was calculated from three independent gels. These values were plotted against the CssR concentration in log_10_ scale, and a sigmoidal fit was performed. The turning point of the curve was defined as the apparent *K*_*D*_ value. **c** Identification of the CssR-binding site within the 131-bp *P*_*cssR*_ promoter using the DNase I footprinting assay

To determine whether CssR directly regulated the expression of its own gene and *ncgl1577*, we examined the direct interaction between purified His_6_-CssR and 131-bp *P*_*cssR*_ using EMSAs. The native molecular mass of the purified His_6_-CssR proteins was found to be 52 kDa by size exclusion chromatography (Additional file 1: Figure S5), indicating a homodimeric structure. This size has also been documented for other members of the TetR family, e.g., TetR [46], CamR [47], and EthR [48]. Incubation of 131-bp *P*_*cssR*_ with His_6_-CssR caused a clear delay in promoter DNA migration, and the abundance of the delayed migration of *P*_*cssR*_ depended on the amount of His_6_-CssR (Fig. 4a). This effect was specific because the combination of His_6_-CssR and 131-bp control DNA fragments amplified from the *cssR* coding region showed no detectable His_6_-CssR binding (Fig. 4a, lane 8); incubation of BSA with 131-bp *P*_*cssR*_ did not lead to retarded mobility (Fig. 4a, lane 9). The apparent *K*_*D*_ value for *P*_*cssR*_ was approximately 17 nM CssR (Fig. 4b), which was within the range found for other transcriptional regulators [19]. These results showed that the CssR-binding site was indeed in the intergenic region between *cssR* and *ncgl1577.* To further identify the precise CssR-binding site, DNase I footprint analysis was performed (Fig. 4c). A protected DNA region extending from − 81 to − 56 bp upstream of the initiation codon of the *cssR* ORF with high affinity for CssR was identified, indicating that the most important part of the CssR-binding site is located within these 26 bp. To confirm the footprint data, a mutated 131-bp promoter DNA sequence (131-bp *P*_*cssRM*_) was used for an EMSA. As shown in Additional file 1: Figure S2c, 131-bp *P*_*cssRM*_ abolished the formation of DNA–protein complexes in the EMSA. Consistently, the mutations in the identified CssR-binding site led to the high β-galactosidase activities of the *cssR* and *ncgl1577* promoters in the WT(pXMJ19)(*P*_*cssRM*_*::lacZY*), WT(pXMJ19)(*P*_*ncgl1577M*_*::lacZY*), Δ*cssR*(pXMJ19-*cssR*)(*P*_*ncgl1577M*_*::lacZY*), and Δ*cssR*(pXMJ19-*cssR*)(*P*_*cssRM*_*::lacZY*) strains, similar to those in the Δ*cssR*(pXMJ19)(*P*_*cssRM*_*::lacZY*) and Δ*cssR*(pXMJ19)(*P*_*ncgl1577M*_*::lacZY*) mutant strains (Additional file 1: Figure S2d). Thus, CssR directly inhibited its own expression and that of the *ncgl1576*-*ncgl1577* operon by virtue of being located downstream of the − 10/35 regions of the proposed promoters, indicating that repression was achieved by inhibition of initiation complex formation. These results further indicated that the corresponding sequence was required for CssR binding.

### Identification of the CssR-binding motif

Inspection of the upstream regions of the *cssR* gene in *C. deserti*, *C. crudilactis*, *C. callunae*, and *C. halotolerans* revealed that they all contain sequence motifs similar to those protected by CssR in *C. glutamicum*. As shown in the alignment presented in Additional file 1: Figure S6a, a putative CssR consensus sequence was derived, and it contained an imperfect inverted repeat: 5′-TAA(G)TGN_13_CA(G)TTA-3′ (25 bp). A binding motif of this type and size are typical of TetR-type transcriptional regulators such as TetR [46] or CamR [47].

To confirm the proposed CssR consensus sequence, EMSAs were performed for mutational analysis. In the experimental results shown in Additional file 1: Figure S6b, the shift of six different DNA fragments, which were amplified by PCR, was analyzed, in each case with excess CssR. Fragments M1–M5 represent derivatives of a WT fragment with mutations within or outside the proposed binding motif. Exchange of the three outer (fragment M1) or the three inner (fragment M2) bases of the imperfect inverted repeat completely inhibited the shift, as did an exchange of the six or seven bases separating the inverted repeats (fragment M3 or M4). In contrast, exchange of four bases outside the proposed binding site (fragment M5) did not prevent the shift. These data provided strong support for the CssR consensus binding site proposed above.

### Expression of the *cssR-ncgl1579* and *ncgl1576-ncgl1577* operons was induced by antibiotics and heavy metals via CssR but not oxidants

To examine whether the expression of the *cssR*-*ncgl1579* and *ncgl1576-ncgl1577* operons respond to xenobiotics at the transcriptional level, qRT-PCR profiling was performed, and the *lacZY* activity of the chromosomal promoter fusion reporter strain was determined. For simplicity, we used GEN, CdCl_2_, H_2_O_2_, and diamide as inducers in the following experiments. As shown in Figs. 2a, e, 3a, e, in the absence of GEN and CdCl_2_, the Δ*cssR*(pXMJ19)(*P*_*cssR*_*::lacZY*) strains exhibited significantly higher *lacZY* activity than the WT(pXMJ19)(*P*_*cssR*_*::lacZY*) or Δ*cssR*(pXMJ19-*cssR*)(*P*_*cssR*_*::lacZY*) strains; the Δ*cssR*(pXMJ19)(*P*_*ncgl1577*_*::lacZY*) strains exhibited significantly higher *lacZY* activity than the WT(pXMJ19)(*P*_*ncgl1577*_*::lacZY*) or Δ*cssR*(pXMJ19-*cssR*)(*P*_*ncgl1577*_*::lacZY*) strains. However, the *lacZY* activities of the *cssR* and *ncgl1577* promoters in the GEN- and CdCl_2_-exposed WT(pXMJ19)(*P*_*cssR*_*::lacZY*) and WT(pXMJ19)(*P*_*ncgl1577*_*::lacZY*) strains were obviously higher than that in the untreated WT(pXMJ19)(*P*_*cssR*_*::lacZY*) or WT(pXMJ19)(*P*_*ncgl1577*_*::lacZY*) strains. The addition of GEN and CdCl_2_ did not change the *lacZY* activity of the *cssR* and *ncgl1577* promoters in the Δ*cssR*(pXMJ19)(*P*_*cssR*_*::lacZY*) and Δ*cssR*(pXMJ19)(*P*_*ncgl1577*_*::lacZY*) strains, which was maintained at the same level as that observed in the Δ*cssR*(pXMJ19)(*P*_*cssR*_*::lacZY*) and Δ*cssR*(pXMJ19)(*P*_*ncgl1577*_*::lacZY*) strains without xenobiotic treatment. Moreover, analysis of the *lacZY* activity showed dose-dependent expression in the WT (pXMJ19) and Δ*cssR* (pXMJ19-*cssR*) strains in response to GEN and CdCl_2_ exposure (Figs. 2a, e, 3a, e). Similar CssR regulatory patterns of *cssR* and *ncgl1577* were also observed at the mRNA transcriptional level through qRT-PCR analysis (Figs. 2b, f, 3b, f). Further analysis at the protein level indicated that similar regulation was observed for the production of NCgl1579 and NCgl1577. In the WT strain, GEN and CdCl_2_ treatment greatly increased NCgl1577 and NCgl1579 at the cellular level, similar to the Δ*cssR* strain in the absence of GEN and CdCl_2_ (Figs. 2c, g, 3c, g; Additional file 1: Figure S7). Interestingly, the transcription of *cssR* and *ncgl1577* was negligibly affected by H_2_O_2_ and diamide (Additional file 1: Figures S8 and S9). These results clearly demonstrated that *cssR* and *ncgl1577* were upregulated in response to increasing antibiotic and heavy metal concentrations, indicating that antibiotics and heavy metals rendered CssR incapable of binding DNA, instigating the transcription of its own gene, *ncgl1579*, and that of the *ncgl1576-ncgl1577* operon.

### The ability of CssR to bind the promoter regions of the *cssR-ncgl1579 *and *ncgl1576-ncgl1577* operons was inhibited by heavy metals and antibiotics but not oxidants

**Fig. 5:**
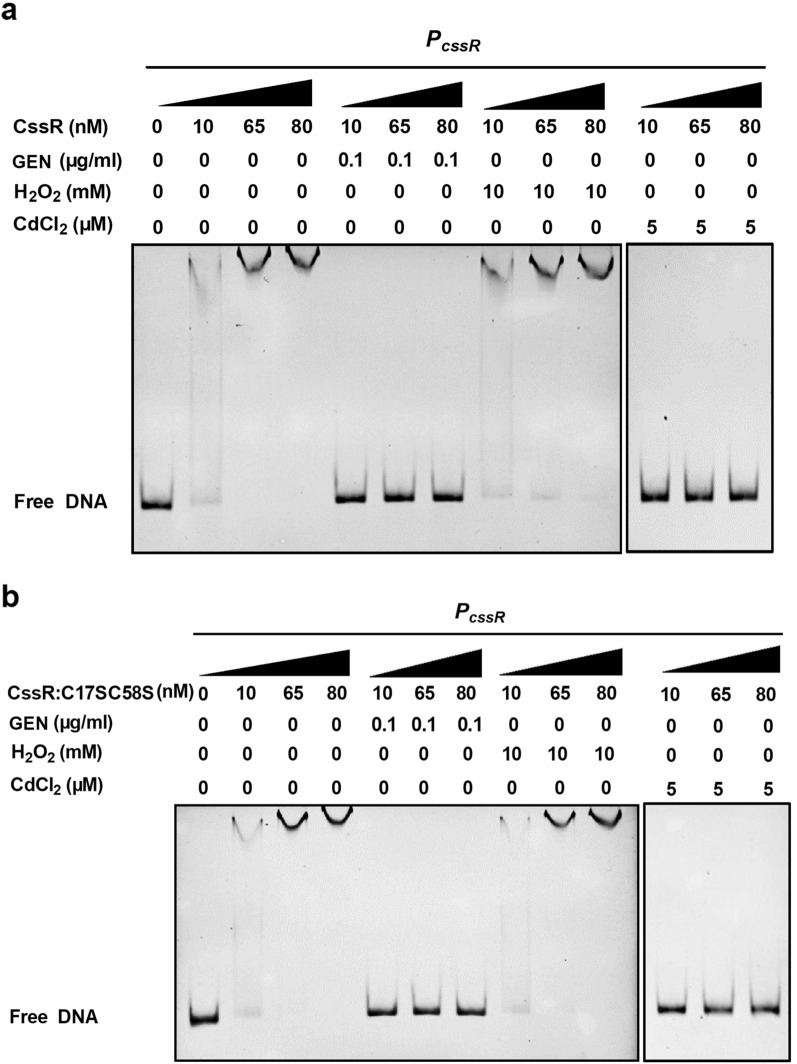
Inhibition of the DNA binding of CssR and CssR:C17SC58S by GEN or CdCl_2_ but not H_2_O_2_. **a** GEN, CdCl_2_, or H_2_O_2_ was added to the binding reaction mixture containing CssR and EMSA was performed. **b** GEN, CdCl_2_, or H_2_O_2_ was added to the binding reaction mixture containing CssR:C17SC58S and EMSA was performed

Interestingly, the binding of His_6_-CssR to *P*_*cssR*_ was prevented by the addition of GEN or CdCl_2_ (Fig. 5a)*.* However, 10 mM H_2_O_2_ did not impair the DNA-binding activity of His_6_-CssR (Fig. 5a). Together, these results showed that CssR specifically recognized operators and then directly bound the *cssR* and *ncgl1577* intergenic regions in a sequence-specific manner. In the presence of GEN or CdCl_2_, CssR dissociated from the promoter DNA, leading to the upregulation of target genes.

**Fig. 6:**
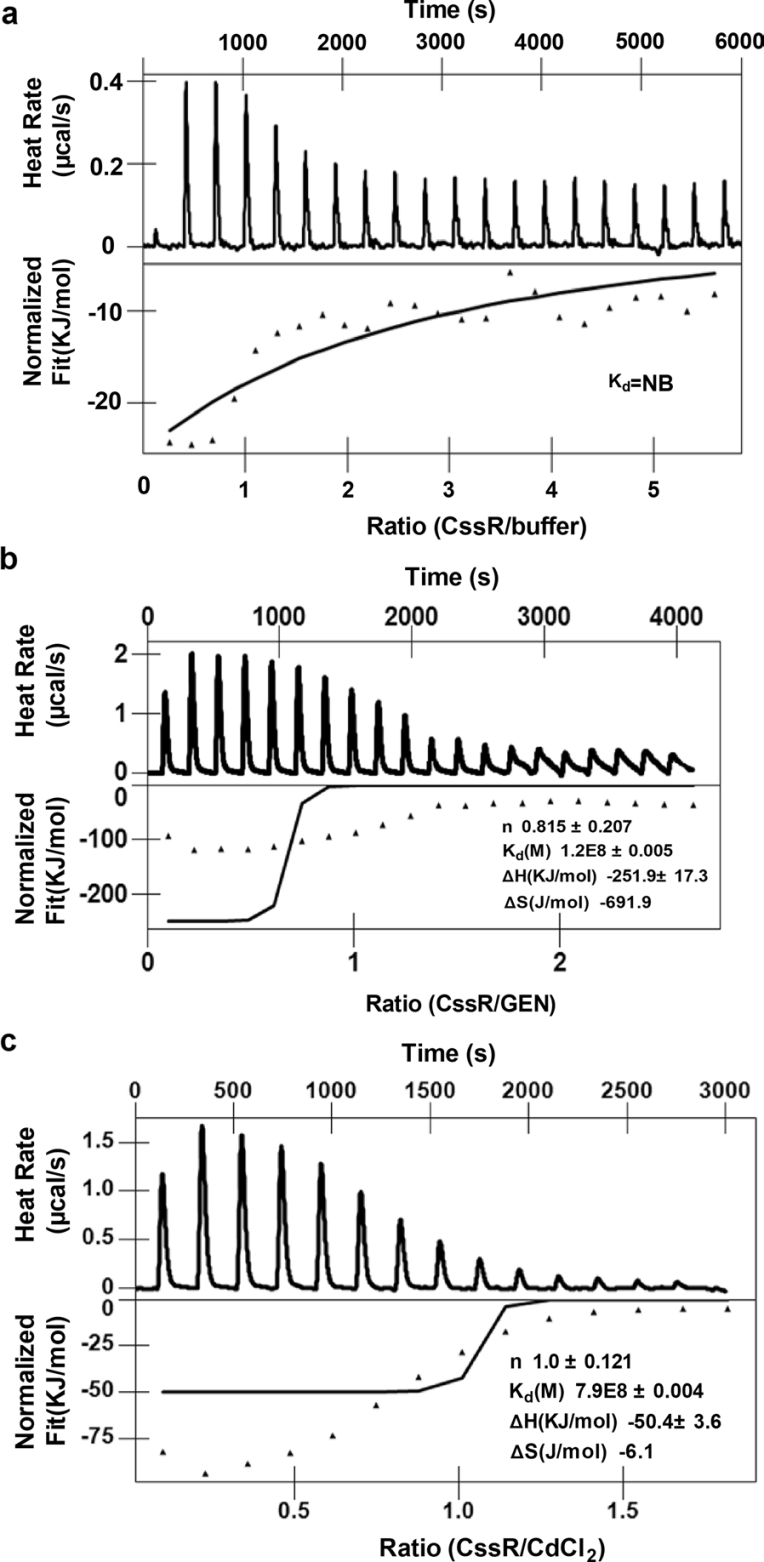
The binding of ligand by CssR. Binding activity of ligand-free CssR to buffer (**a**), GEN (**b**), or CdCl_2_ (**c**) was performed by isothermal titration calorimetry (ITC), respectively. Data were analyzed using the NanoAnalyze software (TA Instruments). Similar results were obtained in three independent experiments, and data shown were from one representative experiment. K_*d*_, dissociation constant. NB, no detectable binding

Therefore, to further investigate whether GEN and CdCl_2_ are ligands of CssR, isothermal titration calorimetry (ITC) analysis was performed. No binding was observed when buffer was titrated into CssR (Fig. 6a). However, the strength of the interaction between CssR and GEN or CdCl_2_ was measured by ITC and possessed negative enthalpic contribution of a typical hyperbolic binding curve (Fig. 6b, c). The stoichiometry N of CssR to GEN or CdCl_2_ was between 0.8 and 1.2, which aligned with the standard 1:1 complex formation between a ligand and protein. Moreover, the enthalpic (ΔH) and entropic (ΔS) parameters of CssR binding to GEN or CdCl_2_ were − 251.9 ± 17.3 kJ mol^−1^ and − 691.9 J mol^−1^, or − 50.4 ± 3.6 kJ mol^−1^ and − 6.1 J mol^−1^, yielding a dissociation constant, *K*_d_, of 0.012 ± 0.005 μM or 0.079 ± 0.004 μM, respectively, indicating that CssR had a high affinity for GEN and CdCl_2_. The *K*_d_ values obtained from *C. glutamicum* CssR were similar to those of known TetR-type *E. coli* regulators and the *Staphylococcus aureus* TetR-type quaternary ammonium compound regulator QacR [46, 49], indicating a high probability that antibiotics and heavy metals bind CssR in vivo. This finding also suggested that CssR can directly bind GEN and CdCl_2_, the outcome of which is CssR dissociation from the target DNA sequence and, hence, target regulon activation.

### Oxidant neither altered the conformation of CssR nor modified its cysteine residues

The amino acid sequence of CssR contains two cysteine residues at positions 17 and 58. Thus, we thought these residues might control target gene expression through the action of versatile posttranslational thiol modification mechanisms. Unexpectedly, CssR incubated with H_2_O_2_, CHP or diamide migrated as a band of approximately 27 kDa on a 15% nonreducing SDS-PAGE gel, which closely corresponded to the sum of the molecular mass (~ 22 kDa) of the native CssR protein as deduced from its amino acid sequence and His_6_ (approximately 5 kDa) (Additional file 1: Figure S10a). This result was confirmed by measuring the thiol content of H_2_O_2_-, CHP- and diamide-treated CssR with a DTNB assay. The DTNB assay showed that the DTT-treated CssR had 1.795 ± 0.075 thiol groups per monomer; H_2_O_2_-, CHP-, and diamide-treated CssR monomers contained 1.810 ± 0.15, 1.839 ± 0.126, or 1.785 ± 0.107 thiol groups, respectively. These results indicated that the thiol contents of CssR were unchanged both before and after exposure to oxidant and that there was no thiol modification upon oxidant treatment (Additional file 1: Figure S10b). Our data indicated that oxidative stress did not influence cssR conformation. In addition, analysis of the transcription levels revealed that in the Δ*cssR*(pXMJ19-*cssR:C17SC58S*) strain, the expression of the *cssR*-*ncgl1579* and *ncgl1576-ncgl1577* operons under GEN and CdCl_2_ stress conditions remained unchanged (Figs. 2 and 3); an EMSA also revealed that CssR:C17SC58S behaved very similarly to CssR (Fig. 5b). Thus, we speculated that CssR did not regulate genes involved in the stress response via a thiol-based mechanism.

### Cellular redox homeostasis-maintaining reducing systems were negatively regulated by CssR

**Fig. 7:**
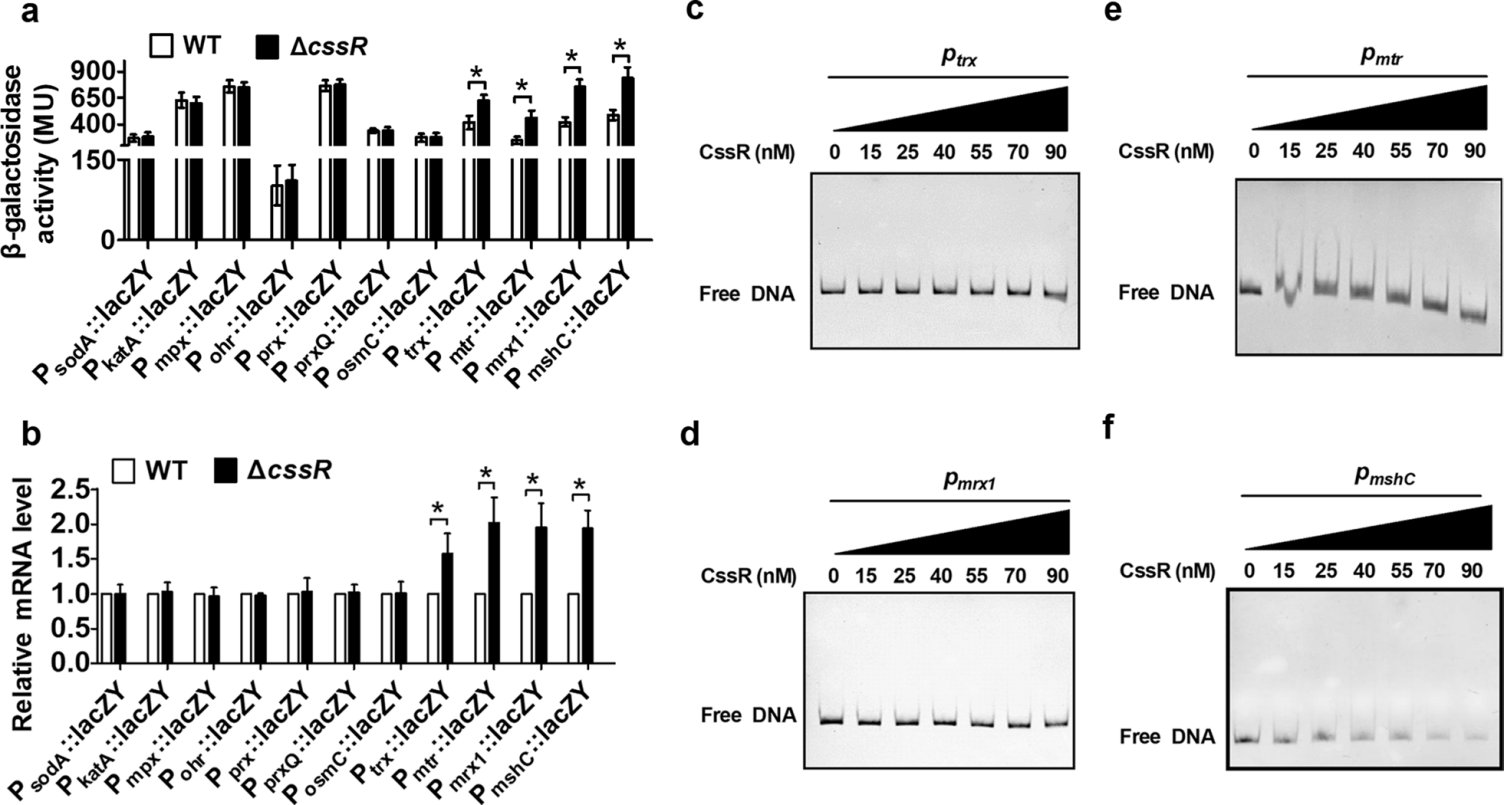
Negative regulation of reduce systems by CssR. **a** β-galactosidase analyses of the different ROS-detoxifying enzymes and the reducing systems promoter activities by using the transcriptional *lacZY* chromosomal fusion reporter expressed in the WT and Δ*cssR* mutant under normal conditions. **b** Quantitative RT-PCR analyses of the different ROS-detoxifying enzymes and the reducing systems expression in WT and Δ*cssR* mutant under indicated conditions. Results were the average of three independent experiments; the standard deviation was indicated by bars. The mRNA levels were presented relative to the value obtained from WT cells. Relative transcript levels of WT strains were set at a value of 1.0. Data shown were the averages of three independent experiments, and error bars indicated the SDs from three independent experiments. *P < 0.05. **c**–**f** CssR did not bind directly to the promoters of the genes involved in redox homeostasis. The interaction between His_6_-CssR and the *trx* promoter fragment (*P*_*trx*_) (**c**), the *mrx1* promoter fragment (*P*_*mrx1*_) (**d**), the *mtr* promoter fragment (*P*_*mtr*_) (**e**), or the *mshC* promoter fragment (*P*_*mshC*_) (**f**)

Oxidant-resistant strains typically have high levels of ROS-detoxifying enzymes. In *C. glutamicum*, SodA, KatA, MPx, Ohr, Prx, PrxQ, and OsmC have been shown to be the major ROS-detoxifying enzymes and to be important for survival under oxidative stress [7–11]. To test whether CssR controlled the expression of ROS-detoxifying enzymes, the *lacZY* activity of a chromosomal promoter fusion reporter-expressing strain and qRT-PCR was determined. The findings showed that the transcription levels of the *sodA*, *katA*, *mpx*, *ohr*, *prx*, *prxQ*, and *osmC* genes in the Δ*cssR* strain were almost the same as those in the WT strain (Fig. 7a, b).

Oxidants have the ability to disturb metabolism, such as carbon metabolism, precursor supply levels, energy metabolism and redox metabolism. Therefore, we postulated that reducing systems might be influenced in the Δ*cssR* strain, and we measured NADPH levels and the transcription of redox homeostasis-related genes. As shown in Additional file 1: Table S3, the NADPH/NADP^+^ ratio in the Δ*cssR* strain (1.29 ± 0.02) was approximately 1.53-fold higher than that in the WT strain (0.84 ± 0.05). In contrast, there was no obvious difference in the NADH/NAD^+^ ratio in the WT and Δ*cssR* strains. This result was consistent with the key roles of NADH and NADPH, which are known to act as a pro-oxidant and antioxidant, respectively [6].

The transcription of the genes *trx*, *mtr*, *mrx1*, and *mshC*, which are key members of three alterative physiological reducing systems in *C. glutamicum*, i.e., the MSH system (MSH/Mtr), Mrx1 system (Mrx1/Mtr/MSH) and Trx system (Trx/TrxR), as determined by the *lacZY* activity of the chromosomal promoter fusion reporter-expressing strain and qRT-PCR, was analyzed. Because Trx and TrxR are cotranscribed [12], we measured the transcription of *trx*. As expected, the transcription levels of the *trx*, *mtr, mrx1*, and *mshC* genes in the Δ*cssR* strain were higher than those in the WT strain. These results indicated that the oxidant-resistant ability of the Δ*cssR* strain might be attributable to increased reducing power levels (Fig. 7a, b).

To test whether CssR directly regulated the expression of the aforementioned genes involved in supplying reducing power, the direct interaction of CssR with the promoters of the genes *trx*, *mrx1*, *mtr*, and *mshC* was subsequently assessed by EMSAs (Fig. 7c–f). Interestingly, CssR did not bind to the promoter regions of the *trx*, *mrx1*, *mtr*, and *mshC* genes. Moreover, a putative CssR consensus sequence was not found in their promoter regions. These results indicated that they were not direct targets of CssR.

## Discussion

In this study, we provide insight into the TetR-type regulator CssR in *C. glutamicum*. Recently, members of the TetR regulator family have attracted considerable attention, as some TetR proteins have been shown to contribute to a wide variety of stress-related toxic compound resistance phenotypes [28]. In several cases, TetR-type regulators have been described to control multidrug transporter genes, often located in the same operon or in immediately adjacent upstream regions but with differing orientations. The resulting phenotype of enhanced drug resistance has been considered a result of increased efflux of a toxic compound. In the present study, we demonstrated that CssR was a transcriptional regulator of the TetR family of genes that repressed the expression of the *ncgl1576*-*ncgl1577* operon and its structural gene. The *ncgl1576*-*ncgl1577* operon, located immediately upstream and in the opposite direction of *cssR*, encodes an ATP-binding cassette (ABC), some of which are involved in the export of a wide range of antimicrobial compounds and have been implicated in the stress response. Through survival assays, we observed notable resistance of the Δ*cssR* strain and high-level susceptibility of Δ*ncgl1576*-*ncgl1577* to antibiotics and heavy metals. Further expression property analyses showed that the expression of *cssR* and *ncgl1577* was induced by antibiotics and heavy metals. Moreover, *cssR* and *ncgl1577* expression was derepressed by inactivation of the transcriptional repressor CssR. Remarkably, although many TetR family transcriptional regulators have been reported to control the expression of genes required for bacteria to adapt to environmental stresses, it is not clear whether most of these regulators bind ligands and the identify of these ligands [45]. Considering our findings showing that CssR has high affinity for antibiotics and heavy metals, we speculated that CssR binding is affected by one or several antibiotics and/or heavy metals, causing cell stress resistance. These data indicated that antibiotic- or heavy metal-binding to CssR directly interfered with the ability of CssR to recognize DNA, which led to *cssR* and *ncgl1577* expression, and then, the product of the *ncgl1576-ncgl1577* operon was increased, and it actively exported toxic compounds.

A sequence alignment assay showed that CssR was conserved in several species in the genus *Corynebacterium*. Notably, the genomic organization of *cssR* in *C. deserti*, *C. crudilactis, C. callunae*, and *C. halotolerans* was almost identical to that in * C. glutamicum*. This indicated that the CssR homologs share a similar regulatory mechanism. Interestingly, a BLAST search also revealed that CssR shared some sequence similarity with the TetR family of bacterial regulator proteins, such as AcrR, EnvR, and KstR. Therefore, we believe that the present study can provide insight into other members of the TetR family of transcriptional regulators that directly bind specific ligands. Our study on the regulatory mechanism of CssR may also lead to a greater understanding of stress response mechanisms involving TetR family transcriptional regulators.

Although the *cssR*-deleted strain (Δ*cssR*) also exhibited increased resistance to oxidants such as H_2_O_2_ and diamide, the apparent failure of *cssR* and *ncgl1577* induction was observed under oxidant treatment. Lack of effects from Cys site-directed mutagenesis of CssR on the *cssR* and *ncgl1577* expression levels, oxidant exposure on the DNA-binding activity of CssR, or oxidant exposure on the morphology of CssR implied that CssR controlled the target genes expression through a mechanism that does not involve cysteine oxidation-based thiol modification. That is, Cys17 and Cys58 played no role in the regulation of CssR activity. Oxidants, such as diamide and H_2_O_2_, have been shown not only to disrupt the cellular redox system but also the defense system against ROS [50]. For example, many antioxidant enzymes remove H_2_O_2_ at the expense of reductants such as NAD(P)H and MSH. Diamide specifically oxidized thiol groups such as those in cysteine, resulting in the accumulation of nonnative disulfide bonds [6], which were repaired via the repair reducing system. Interestingly, Mailloux et al. found that metabolism played a key role in *Pseudomonas* species defenses against oxidative stress [6]. Under H_2_O_2_ conditions, the intracellular concentrations of H_2_O_2_-scavenging metabolic intermediates and the antioxidant NADPH were increased. Recently, Hong et al. found that TetR-type OsrR in *C. glutamicum* was not induced by H_2_O_2_. However, it was involved in H_2_O_2_ resistance, strongly affecting the cellular ratio of NADPH/NADP^+^, and exhibited a regulatory role for redox homeostasis-related genes, such as *trx* and *mtr* [25]. Therefore, we suggested that CssR, despite its moderate sequence similarity to OsrR (approximately 29% identity), may be associated with metabolism, detoxification proteins and repressed system repair. The MSH system (MSH/Mtr), Mrx1 system (Mrx1/Mtr/MSH) and Trx system (Trx/TrxR) have been shown to reduce disulfides in oxidized proteins to maintain intracellular thiol-disulfide homeostasis during bursts of oxidative stress [12, 51, 52]. MSH reportedly acts as a redox buffer and is essential for cellular defenses against ROS and maintaining the reducing state of the cytoplasm [1]. Moreover, MshC was previously found to be necessary for synthesizing MSH, and Δ*mshC* mutants lost the ability to produce MSH [1]. Thus, their expression level could reflect the intracellular MSH content to a certain extent. As shown in Fig. 7, genes such as *trx*, *mrx1*, *mtr*, and *mshC* showed increased transcription in the Δ*cssR* strain, and the NADPH/NADP^+^ ratio was higher in the Δ*cssR* strain. However, detoxification proteins, such as *sodA*, *katA*, *mpx*, *prx*, *prxQ*, *osmC*, and *ohr*, were not upregulated in the Δ*cssR* mutant. In *C. glutamicum*, *mpx*, *prx*, *prxQ*, *osmC*, and *ohr* encode peroxidases whose activities are regenerated by Trx, TrxR, Mrx1, Mtr, and MSH [7–11]. Therefore, the protective roles of the genes involved in redox homeostasis in the Δ*cssR* mutant strain upon oxidant challenge were not realized by supporting peroxidase activity. Additionally, the impact of the enhanced production of redox homeostasis-related genes on oxidative damage restoration should not be very high in the Δ*cssR* mutant, which may be proven by the oxidant-resistant phenotype of the Δ*cssR* mutant and indirectly by CssR reliance on redox homeostasis-related genes to a certain extent. Combining these data, we suggested that there might be some oxidant-scavenging metabolic intermediates that act as substrates of CssR, which indirectly caused it to regulate the reduction system. To our surprise, the phenotype of the *cssR* mutant was almost opposite to that of the *osrR* mutant. The *osrR* gene was found to play a positive role in H_2_O_2_-detoxifying metabolic systems, except for catalase [25]. Thus, it will be necessary to elucidate how the *cssR* and *osrR* genes collaborate with each other to regulate genes involved in oxidative stress responses.

To date, members of the well-known TetR family have been found to form homodimers and then bind to the palindromic sequences of the target gene promoter regions, acting as transcriptional repressors [28]. Our results showed that CssR recognized the 25-bp operator, which has an imperfect palindromic sequence [TAA(G)TGN_3_GN_5_CN_3_CA(G)TTA]. Since, in addition, we found that CssR occurred as a homodimer in its native form, we proposed that CssR binds to the target gene promoter as a homodimer.

Overall, CssR is a TetR family repressor that plays a critical role in the bacterial response to stresses by enhancing the expression of ABC and reductants with ligand-mediated attenuation of DNA binding but not cysteine oxidation-based thiol modifications.

## Supplementary Information

**Additional file 1: Table S1.** Bacterial strains and plasmids used in this study. **Table S2.** Primers used in this study. **Table S3.** Ratios of NADPH/NADP^+^ and NADH/NAD^+^ in *C. glutamicum* strains. **Figure S1.** Multiple sequence alignment. **Figure S2.** Detailed genetic maps of the regulatory region of CssR. **Figure S3.** Growth curves of the WT strain (the *C. glutamicum* RES167 parental strain), Δ*cssR* mutant (the mutant lacking *cssR*), and Δ*ncgl1576-ncgl1577* mutant (the mutant lacking *ncgl1576-ncgl1577*) under normal conditions. **Figure S4.** 91-bp *cssR* transcript (corresponding to nucleotides + 1 to + 91 relative to the translational start codon (ATG) of *cssR* gene) was amplified from the remaining *cssR* ORF in Δ*cssR* mutant with primers QcssR-F and QcssR-R. **Figure S5.** Purification of His_6_-CssR and determination of the native molecular mass by size exclusion chromatography. **Figure S6.** Sequence of the *cssR* promoter region of *C. glutamicum* aligned to putative *cssR* promoter regions from other *Corynebacterium* species. **Figure S7.** The NCgl1577 and NCgl1579 were examined in *C. glutamicum*. **Figure S8.** Negative regulation of cssR by CssR. **Figure S9.** Negative regulation of *ncgl1577* by CssR. **Figure S10.** Redox response of CssR in vitro.

## Data Availability

All the data generated or analyzed during this study are included in the manuscript and its additional file.

## Abbreviations

MSH: Mycothiol
TetR: Tetracycline repressor
ABC: ATP-binding cassette
ROS: Reactive oxygen species
DTNB: 5,5′-Dithio-bis(2-nitrobenzoic acid)
EMSA: Electrophoretic mobility shift assay
CHP: Cumene hydroperoxide
H_2_O_2_: Hydrogen peroxide
DTT: Dithiothreitol
CBB: Coomassie Brilliant Blue
β-ME: β-Mercaptoethanol
ONPG: *O*-Nitrophenyl-β-d-galactopyranoside
CFU: Colony-Forming Unit
ITC: Isothermal titration calorimetry
GEN: Gentamicin
ERY: Erythromycin
CIP: Ciprofloxacin
*t*-BHP: *tert*-Butyl hydroperoxide
IPTG: Isopropyl β-d-1-thiogalactopyranoside
PVDF: Polyvinylidene fluoride
NemR: *N*-Ethylmaleimide regulator
OsrR: Oxidative stress response regulator
