# Supplementary material for: The cssR gene of Corynebacterium glutamicum plays a negative regulatory role in stress responses
Source: Microb Cell Fact. 2021 Jun 3;20:110. doi: 10.1186/s12934-021-01600-8 (PMC8176726; doi:10.1186/s12934-021-01600-8)
Supplement: Supplementary file 1 — Additional file 1: Table S1. Bacterial strains and plasmids used in this study. Table S2. Primers used in this study. Table S3. Ratios of NADPH/NADP+ and NADH/NAD+ in C. glutamicum strains. Figure S1. Multiple sequence alignment. Figure S2. Detailed genetic maps of the regulatory region of CssR. Figure S3. Growth curves of the WT strain (the C. glutamicum RES167 parental strain), ΔcssR mutant (the mutant lacking cssR), and Δncgl1576-ncgl1577 mutant (the mutant lacking ncgl1576-ncgl1577) under normal conditions. Figure S4. 91-bp cssR transcript (corresponding to nucleotides + 1 to + 91 relative to the translational start codon (ATG) of cssR gene) was amplified from the remaining cssR ORF in ΔcssR mutant with primers QcssR-F and QcssR-R. Figure S5. Purification of His6-CssR and determination of the native molecular mass by size exclusion chromatography. Figure S6. Sequence of the cssR promoter region of C. glutamicum aligned to putative cssR promoter regions from other Corynebacterium species. Figure S7. The NCgl1577 and NCgl1579 were examined in C. glutamicum. Figure S8. Negative regulation of cssR by CssR. Figure S9. Negative regulation of ncgl1577 by CssR. Figure S10. Redox response of CssR in vitro. [file 12934_2021_1600_MOESM1_ESM.docx]

**Additional file 1**

**The *cssR* gene of *Corynebacterium glutamicum* plays a negative regulatory role in stress responses**

Yang Liu ^#a^, Wenzhi Yang^#c^, Tao Su^a^, Chengchuan Che^a^, Guizhi Li ^1^, Can Chen^b*^, Meiru Si^a*^

^a^ College of Life Sciences, Qufu Normal University, Qufu, Shandong 273165, China;

^b^ Key Laboratory of Plant Genetics and Molecular Breeding, Henan Key Laboratory of Crop Molecular Breeding & Bioreactor, College of Life Science and Agronomy, Zhoukou Normal University, Zhoukou, Henan 466001, China

^c^ School of Food Science and Nutrition, University of Leeds, Leeds LS2 9JT, UK

**Running title:** Stress-sensing TetR-type sensor CssR of *C. glutamicum*

**^#^** These authors contributed equally to this work.

**^*^** Corresponding authors:

Meiru Si, Can Chen

E-mail: [smr1016@126.com](mailto:smr1016@126.com); chenc02@126.com

Tel: 86-15666758564

**Table S1.** Bacterial strains and plasmids used in this study.

| **Strains or plasmids** | **Relevant genotype description** | **References** | |
| --- | --- | --- | --- |
| **Strains** | | | |
| ***Corynebacterium glutamicum*** | | | |
| RES167 | Restriction-deficient mutant of ATCC13032, Δ(*cglIM-cglIR-cglIIR*) | | [1] |
| Δ*cssR* | *cssR* deleted in RES167 | | This study |
| Δ*ncgl1576-ncgl1577* | *ncgl1576* and *ncgl1577* deleted in RES167 | | This study |
| WT(pXMJ19) | RES167 parental strain containing pXMJ19 | | [2] |
| Δ*cssR*(pXMJ19) | Δ*cssR* containing pXMJ19 | | This study |
| Δ*ncgl1576-ncgl1577*(pXMJ19) | Δ*ncgl1576-ncgl1577* containing pXMJ19 | | This study |
| Δ*cssR*(pXMJ19-*cssR*) | Δ*cssR* containing pXMJ19-*cssR* | | This study |
| Δ*cssR*(pXMJ19-*cssR:C17SC158S*) | Δ*cssR* containing pXMJ19-*cssR:C17SC158S* | | This study |
| Δ*ncgl1576-ncgl1577*(pXMJ19-*ncgl1576*-*ncgl1577*) | Δ*ncgl1576-ncgl1577* containing pXMJ19-*ncgl1576*-*ncgl1577* | | This study |
| WT(pXMJ19)(*P_katA_::lacZY*) |  | | [3] |
| WT(pXMJ19)(*P_sodA_::lacZY*) |  | | This study |
| WT(pXMJ19)(*P_mpx_::lacZY*) |  | | [4] |
| WT(pXMJ19)(*P_prx_::lacZY*) |  | | [4] |
| WT(pXMJ19)(*P_prxQ_::lacZY*) |  | | [5] |
| WT(pXMJ19)(*P_osmC_::lacZY*) |  | | [6] |
| WT(pXMJ19)(*P_ohr_::lacZY*) |  | | [7] |
| WT(pXMJ19)(*P_trx_::lacZY*) |  | | [8] |
| WT(pXMJ19)(*P_mrx1_::lacZY*) |  | | [3] |
| WT(pXMJ19)(*P_mtr_::lacZY*) |  | | [9] |
| WT(pXMJ19)(*P_mshC_::lacZY*) |  | | This study |
| Δ*cssR*(pXMJ19)(*P_katA_::lacZY*) |  | | This study |
| Δ*cssR*(pXMJ19)(*P_sodA_::lacZY*) |  | | This study |
| Δ*cssR*(pXMJ19)(*P_mpx_::lacZY*) |  | | This study |
| Δ*cssR*(pXMJ19)(*P_prx_::lacZY*) |  | | This study |
| Δ*cssR*(pXMJ19)(*P_prxQ_::lacZY*) |  | | This study |
| Δ*cssR*(pXMJ19)(*P_osmC_::lacZY*) |  | | This study |
| Δ*cssR*(pXMJ19)(*P_ohr_::lacZY*) |  | | This study |
| Δ*cssR*(pXMJ19)(*P_trx_::lacZY*) |  | | This study |
| Δ*cssR*(pXMJ19)(*P_mrx1_::lacZY*) |  | | This study |
| Δ*cssR*(pXMJ19)(*P_mtr_::lacZY*) |  | | This study |
| Δ*cssR*(pXMJ19)(*P_mshC_::lacZY*) |  | | This study |
| ***E. coli*** | | | |
| BL21(DE3) | Host for expression vector pET28a | | Novagen |
| JM109 | *recA1 supE44 endA1 hsdR17 gyrA96 relA1 thi* Δ(*lac-proAB*)F′(*traD36 proABlacI*^q^ *lacΔZM15*) | | Stratagene |
| **Plasmids** | | | |
| pK18*mobsacB* | Suicide plasmid carrying *sacB* for selecting double crossover in *C. glutamicum*, Km^r^ | | [10] |
| pK18*mobsacB-*Δ*cssR* | Construct used for in-frame deletion of *cssR* | | This study |
| pK18*mobsacB*-*P_cssR_::lacZY* | *P_cssR_::lacZY* fusion in pK18*mobsacB* | | This study |
| pK18*mobsacB*-*P_cssRM_::lacZY* | *P_cssRM_::lacZY* fusion in pK18*mobsacB* | | This study |
| pK18*mobsacB*-*P_ncgl1577_::lacZY* | *P_ncgl1577_::lacZY* fusion in pK18*mobsacB* | | This study |
| pK18*mobsacB*-*P_ncgl1577M_::lacZY* | *P_ncgl1577M_::lacZY* fusion in pK18*mobsacB* | | This study |
| pK18*mobsacB*-*P_mshC_::lacZY* | *P_mshC_::lacZY* fusion in pK18*mobsacB* | | This study |
| pK18*mobsacB*-*P_sodA_::lacZY* | *P_sodA_::lacZY* fusion in pK18*mobsacB* | | This study |
| pXMJ19 | Shuttle vector (*Ptac lacI^q^ pBL1 oriV_C. glutamicum_* pK18 *oriV_E. coli_*) | | [11] |
| pXMJ19-*cssR* | *cssR* cloned into pXMJ19 for complementation | | This study |
| pXMJ19-*cssR:C17SC58S* | *cssR:C17SC58S* cloned into pXMJ19 for complementation | | This study |
| pXMJ19-*ncgl1576-ncgl1577* | *ncgl1576-ncgl1577* cloned into pXMJ19 for complementation | | This study |
| pET28a | Expression vector with N-terminal hexahistidine affinity tag | | Novagen |
| pET28a*-cssR* | *cssR* in pET28a | | This study |
| pET28a*-cssR:C17S* | *cssR:C17S* in pET28a | | This study |
| pET28a*-cssR:C17SC58S* | *cssR:C17SC58S* in pET28a | | This study |
| pET28a*-ncgl1577* | *ncgl1577* in pET28a | | This study |
| pET28a*-ncgl1579* | *ncgl1579* in pET28a | | This study |

**Additional References**

1. Schäfer A, Tauch A, Jager W, Kalinowshi J, Thierbach G, Pühler A. Small mobilizable multi-purpose cloning vectors derived from the *Escherichia coli* plasmids pK18 and pK19: selection of defined deletions in the chromosome of *Corynebacterium glutamicum*. Gene. 1994; 145: 69-73.

2. Jakoby M, Ngouoto-Nkili CE, Burkovski A. Construction and application of new *Corynebacterium glutamicum* vectors. Biotechnol Tech. 1999; 13: 437-441.

3. Li X, Liu Y, Zhong J, Che C, Gong Z, Si M, Yang G. Molecular mechanisms of Mycoredoxin-1 in resistance to oxidative stress in *Corynebacterium glutamicum*. J Gen Appl Microbiol. 2020; doi: 10.2323/jgam.2020.03.002.

4. Si M, Wang T, Pan J, Lin J, Chen C, Wei Y, Lu Z, Wei G, Shen X. Graded Response of the Multifunctional 2-Cysteine Peroxiredoxin, CgPrx, to Increasing Levels of Hydrogen Peroxide in *Corynebacterium glutamicum*. Antioxid Redox Signal. 2017; 26: 1-14.

5. Su T, Si M, Zhao Y, Liu Y, Yao S, Che C, Chen C. A thioredoxin-dependent peroxiredoxin Q from *Corynebacterium glutamicum* plays an important role in defense against oxidative stress. PLoS One. 2018; 13: e0192674.

6. Si M, Su T, Chen C, Wei Z, Gong Z, Li G. OsmC in *Corynebacterium glutamicum* was a thiol-dependent organic hydroperoxide reductase. Int J Biol Macromol. 2019; 136: 642-652.

7. Si M, Wang J, Xiao X, Guan J, Zhang Y, Ding W, Chaudhry MT, Wang Y, Shen X. Ohr protects *Corynebacterium glutamicum* against organic hydroperoxide induced oxidative stress. PLoS one. 2015; 10: e0131634.

8. Che C, Su T, Sun P, Li G, Liu J, Wei Z, Yang G. Thioredoxin and protein-disulfide isomerase selectivity for redox regulation of Proteins in *Corynebacterium glutamicum*. J Gen Appl Microbiol. 2020; 66: 245-255.

9. Si M, Zhao C, Zhang B, Wei D, Chen K, Yang X, Xiao H, Shen X. Overexpression of Mycothiol Disulfide Reductase Enhances *Corynebacterium glutamicum* Robustness by Modulating Cellular Redox Homeostasis and Antioxidant Proteins under Oxidative Stress. Sci Rep. 2016; 6: 29491.

10. Karimova G, Pidoux J, Ullmann A, Ladant D. A bacterial two-hybrid system based on a reconstituted signal transduction pathway. Proc Natl Acad Sci U S A. 1998; 95: 5752-5756.

11. Shen XH, Jiang CY, Huang Y, Liu ZP, Liu SJ. Functional identification of novel genes involved in the glutathione-independent gentisate pathway in *Corynebacterium glutamicum*. Appl Environ Microbiol. 2005; 71: 3442-3452.

**Table S2.** Primers used in this study.

| **Primers** | **5’-3’ sequence** |  |
| --- | --- | --- |
| C_cssR_-F | CGCGGATCCGACTCTAGAGGATCCAAAGGAGGACAACCGTGTCTACAGATCCAGAAGAGTTCG (*Bam*HI) | For cloning *cssR* into pXMJ19 |
| C_cssR_-R | CCGGAATTCCAGCCAAGCTGAATTCTTAGGGGCGGGGCCACTTCATATCG (*Eco*RI) |  |
| C_ncgl1576-ncgl1577_-F | AAAACTGCAGATGAATACCCCTCTTTTGAGAAGC (*Pst*I) | For cloning *ncgl1576-ncgl1577* into pXMJ19 |
| C_ncgl1576-ncgl1577_-R | TTAGACCTTCAGTTCTGGGTGG (*Xba*I) |  |
| O_cssR_-F | CCGGAATTCATGGGTCGCGGATCCGAATTCATGAGAGCAGACGCACTAAAACG (*Eco*RI) | For cloning *cssR* and its variants into pET28a |
| O_cssR_-R | GGACTCGAGGTGGTGGTGGTGGTGCTCGAGTCAGGACTTTCCGTCATGTTCTC (*Xho*I) |  |
| O_ncgl1577_-F | CCGGAATTCATGAATACCCCTCTTTTGAG (*Eco*RI)  AAGC | For cloning *ncgl1577* into pET28a  For cloning *csnR* and its variants into pET28a |
| O_ncgl1577_-R | GGACTCGAGTTAGTTTCCAGCCCCCGTAATG (*Xho*I) |  |
| O_ncgl1579_-F | CGCGGATCATGACGGAAAGTCCTGATCT (*Bam*HI)  AG | For cloning *ncgl1579* into pET28a |
| O_ncgl1579_-R | ACGCGTCGACCTAATAGATTTTGAGCATGTCC (*Sal*I) |  |
| D_ncgl1576-ncgl1577_-F1 | CGCGGATCCAGGGCGGAGACCTCGTCGGGGAG (*Bam*HI) | To generate pK18*mobsacB-*Δ *ncgl1576*-*ncgl1577* |
| D_ncgl1576-ncgl1577_-R1 | GCATGCGGCCGGAAAGTACCAAAG |  |
| D_ncgl1576-ncgl1577_-F2 | CTTTGGTACTTTCCGGCCGCATGCGCATTCTTCCACTGGTTCCACACC |  |
| D_ncgl1576-ncgl1577_*-*R2 | ACGCGTCGACGCCTTGTTGTTGGCGGGTGCGGGC (*Sal*I) |  |
| D_cssR_-F1 | CCGGAATTCCTATGACATGATTACGAATTCGTAAAAATGCCATTAGT TTCCAG (*Eco*RI) | To generate pK18*mobsacB-*Δ*cssR* |
| D_cssR_-R1 | GTTTTCCATGGTGAGCGAATCGTGATGTGTTCGC |  |
| D_cssR_-F2 | CATGGAAAACGGTCTTAAACACGGCATGATGGC |  |
| D_cssR_*-*R2 | CGCGGATCCAGGTCGACTCTAGAGGATCCCCAAACTAGCAGTTTTTAATGGATG (*Bam*HI) |  |
| *P_cssR_*-F | TCCCCCGGGGTGTCGCGGATGGAGAGCCCAG (*Sma*I) | To generate pK18*mobsacB*-*P_cssR_*::*lacZY* |
| *P_cssR_*-R | GTCTGCTCTCATGTTGTTGTCC |  |
| lacZY-F1 | GGACAACAACATGAGAGCAGACACTAGTACTAGTATGACCATGATTACGGATTC(*Spe*I) |  |
| lacZY-R | AAAACTGCAGTTAAGCGACTTCATTCACCTG(*Pst*I) |  |
| *P_ncgl1577_*-F | TCCCCCGGGCAGGTTGTGGTTATGATGTGTTC (*Sma*I) | To generate pK18*mobsacB*-*P_ncgl1577_*::*lacZY* |
| *P_ncgl1577_*-R | AGGCCAAGATTGCATGGGTTTTACTC |  |
| lacZY-F2 | GAGTAAAACCCATGCAATCTTGGCCTACTAGTACTAGTATGACCATGATTACGGATTC(*Spe*I) |  |
| *P_mshC_*-F | TCCCCCGGGGCAGGTCTGCTCGTGATGCTGCTG (*Sma*I) | To generate pK18*mobsacB*-*P_mshC_*::*lacZY* |
| *P_mshC_*-R | AGGGGTATTCATGTATCTTCTTC |  |
| lacZY-F3 | GAAGAAGATACATGAATACCCCT ACTAGTACTAGTATGACCATGATTACGGATTC(*Spe*I) |  |
| *P_sodA_*-F | TCCCCCGGGGCGGGCAACAAGCTGCTCGAGGGAAATC (*Sma*I) | To generate pK18*mobsacB*-*P_sodA_*::*lacZY* |
| *P_sodA_*-R | GTATACAGCCATGGGTAAAAAATCC |  |
| lacZY-F4 | GGATTTTTTACCCATGGCTGTATACACTAGTACTAGTATGACCATGATTACGGATTC(*Spe*I) |  |
| QcssR-F | ATGAGAGCAGACGCACTAAAAC | RT-PCR |
| QcssR-R | CCATGGTGAGCGAATCGTGATG |  |
| Qncgl1577-F | CTTTGGTACTTTCCGGCCGCATGC | RT-PCR |
| Qncgl1577-R | GAATCGATTTCAGGGATGCCCGCC |  |
| EcssR-F | ATGGAGAGCCCAGAGCTTCTC | To produce 131-bp EMSA *cssR* promoter DNA |
| EcssR-R | GTTGTTGTCCTCTAGTTGACAG |  |
| PcssR-footprinting-F | GTGAGTCCGCTGTCTGGAGCTATC | CssR Footprint |
| PcssR-footprinting-R | CGTTTTAGTGCGTCTGCTCTCATG |  |
| CssR-C17S-F | CATCATAACCACAACC*A*GCAATCTCTACCGCAC | To generate *cssR:C17S* DNA fragment |
| CssR-C17S-R | GTGCGGTAGAGATTGC*T*GGTTGTGGTTATGATG |  |
| CssR-C58S-F | CACACTGGACATGGCA*A*GCGCCCAATACCTTTTC | To generate *cssR:C58S* DNA fragment |
| CssR-C58S-R | GAAAAGGTATTGGGCGC*T*TGCCATGTCCAGTGTG |  |
| pET28aMCS-F | GATCTTCCCCATCGGTGATGTC |  |
| QkatA-F | GGACGTATCCGAATACACCAAG | RT-PCR |
| QkatA-R | CTCACCAGCAACAGTAGAGAAG |  |
| QsodA-F | GTCCAAGAACCTTGCATTCAAC | RT-PCR |
| QsodA-R | GCCGAGTGCTGCGGAGTTGAAG |  |
| Qmpx-F | GAAGGGCTTCAAAAACTGTATG | RT-PCR |
| Qmpx-R | TCGTACTGATTTTGCGCGAAAG |  |
| Qprx-F | GACTTCCAGGGCCGCAAGCTTG | RT-PCR |
| Qprx-R | GAATGGAAGATCCTTGGAGATG |  |
| QprxQ-F | CCAAAGCCAACACCCCAGGTTG | RT-PCR |
| QprxQ-R | GGTCCTCACGGAACTTCACCAG |  |
| QosmC-F | GCAGGCCGCTGTTGTTGGCTGC | RT-PCR |
| QosmC-R | CTTGCAACGTGTACAGCAGATC |  |
| Qohr-F | GGATCCGGTGAAGGCACCAATC | RT-PCR |
| Qohr-R | GCCCGATGCTAACTCGGGCACC |  |
| Qtrx-F | CATCGTTGACTTCTGGGCAG | RT-PCR |
| Qtrx-R | CGACGCTGGCAACGACTGCC |  |
| Qmrx1-F | CGACGGCCAAGAGTACGACCTC | RT-PCR |
| Qmrx1-R | CGGAGTAGCGCACGGTTGGGAC |  |
| Qmtr-F | CGACAAGCGCATCGACCTCATC | RT-PCR |
| Qmtr-R | GCTGTTCTTGGCCGGCAATACC |  |
| QmshC-F | ACGCAGCGACCATGGCTGAG | RT-PCR |
| QmshC-R | CGAATGGGGATTCCCAGCTTG |  |
| Emtr-F | AACACCGCGAATTATAGACTG | [1] |
| Emtr-R | GATGTGCTCCTAATTTAGAAATC |  |
| Etrx-F | GAAGTTTTTCAAAGTGTCTGAC | [2] |
| Etrx-R | GTTTCCCTCAAAGTAGTAAACG |  |
| Emrx1-F | GATCACCGCATGTGAAACAAGC | [3] |
| Emrx1-R | GATGGCGCTATGCCGCAAAGATC |  |
| EmshC-F | CCTGTACATCTTAAAAATCCGC | [2] |
| EmshC-R | CGTAATTATTTCCGCGACAAGC |  |
| 16S rRNA-F | ACCTGGAGAAGAAGCACCG | RT-PCR |
| 16SrRNA-R | TCAAGTTATGCCCGTATCG |  |
| Control-F | CTCTACCGCACACACCATCACG | To produce 131-bp control EMSA *cssR* promoter DNA |
| Control-R | GGTATTGGGCGCATGCCATGTC |  |

Underlined sites indicate restriction enzyme cutting sites added for cloning. Letters in italic denote the mutation sites in overlap PCR for site-directed mutation.

**Additional References**

1. Si M, Zhao C, Zhang B, Wei D, Chen K, Yang X, Xiao H, Shen X. Overexpression of Mycothiol Disulfide Reductase Enhances *Corynebacterium glutamicum* Robustness by Modulating Cellular Redox Homeostasis and Antioxidant Proteins under Oxidative Stress. Sci Rep. 2016; 6: 29491.

2. J Busche T, Silar R, Picmanova M, Patek M, Kalinowski J. Transcriptional regulation of the operon encoding stress-responsive ECF sigma factor SigH and its anti-sigma factor RshA, and control of its regulatory network in *Corynebacterium glutamicum*. BMC Genomics. 2012; 13: 445.

3. Li X, Liu Y, Zhong J, Che C, Gong Z, Si M, Yang G. Molecular mechanisms of Mycoredoxin-1 in resistance to oxidative stress in *Corynebacterium glutamicum*. J Gen Appl Microbiol. 2020; doi: 10.2323/jgam.2020.03.002.

**Table S3 Ratios of NADPH/NADP^+^ and NADH/NAD^+^ in *C. glutamicum* strains**

| *C. glutamicum* RES167 | NADPH/NADP^+^ | NADH/NAD^+^ |
| --- | --- | --- |
| Parental strains (WT) | 0.840.05 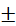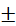 | 0.150.01 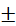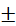 |
| Δ*cssR* mutant | 1.290.02 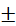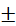 | 0.170.03 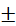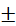 |


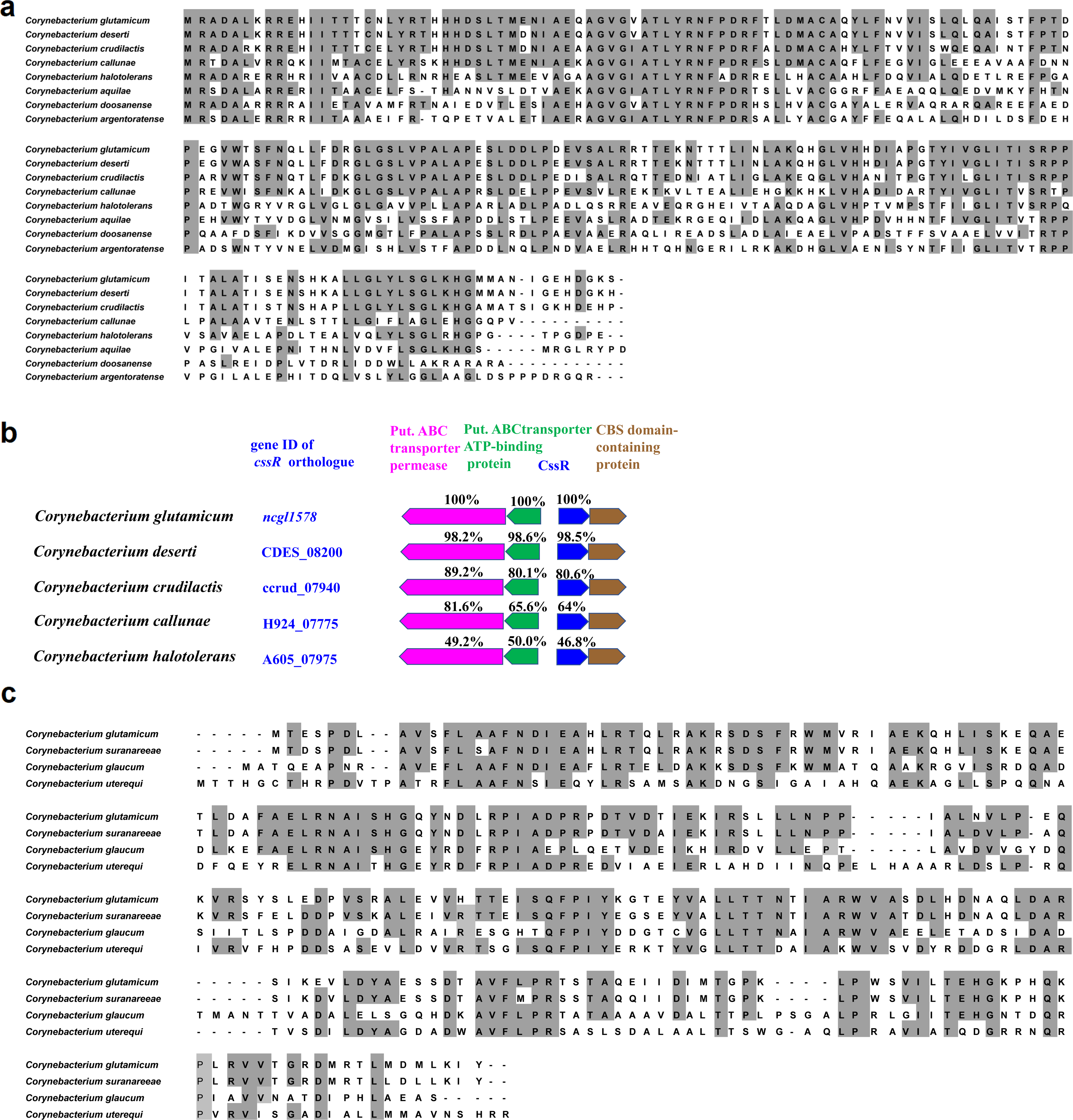


**Figure S1 Multiple sequence alignment. (a)** Multiple sequence alignment of CssR with TetRs from several species of the genera *Corynebacterium.* Residues that were identical in all or at least 4 of the 8 sequences were depicted on the gray background. Reference sequences were retrieved from the NCBI Database, including *C. glutamicum* ATCC CssR (NP_600854), *C. deserti* TetR (ALC06045); *C. crudilactis* TetR (ANE05338); *C. callunae* TetR (AGG66997); *C. halotolerans* TetR (AGF72597); *C. aquilae* TetR (APT84862); *C. doosanense* TetR (AIT61191); *C. argentoratense* TetR (AGU15206). (b) Genomic organization of the *cssR* gene in several *corynebacterial* species. Amino acid sequence identities to the *C. glutamicum* corresponding protein orthologs were given on the column. The genomic context of *C. glutamicum* proteins were extracted from microbesonline (http://microbesonline.org). **(c)** Multiple sequence alignments of NCgl1579 with CBS domain-containing proteins from other organisms*.* Residues that were identical in all or at least 3 of the 4 sequences were depicted on the gray background. Reference sequences were retrieved from the NCBI Database, including *C. glutamicum* NCgl1579 (NP_600855); *C. suranareeae* protein (BAU95992); *C. glaucum* protein (AQQ15356); *C. uterequi* protein (AKK11218).


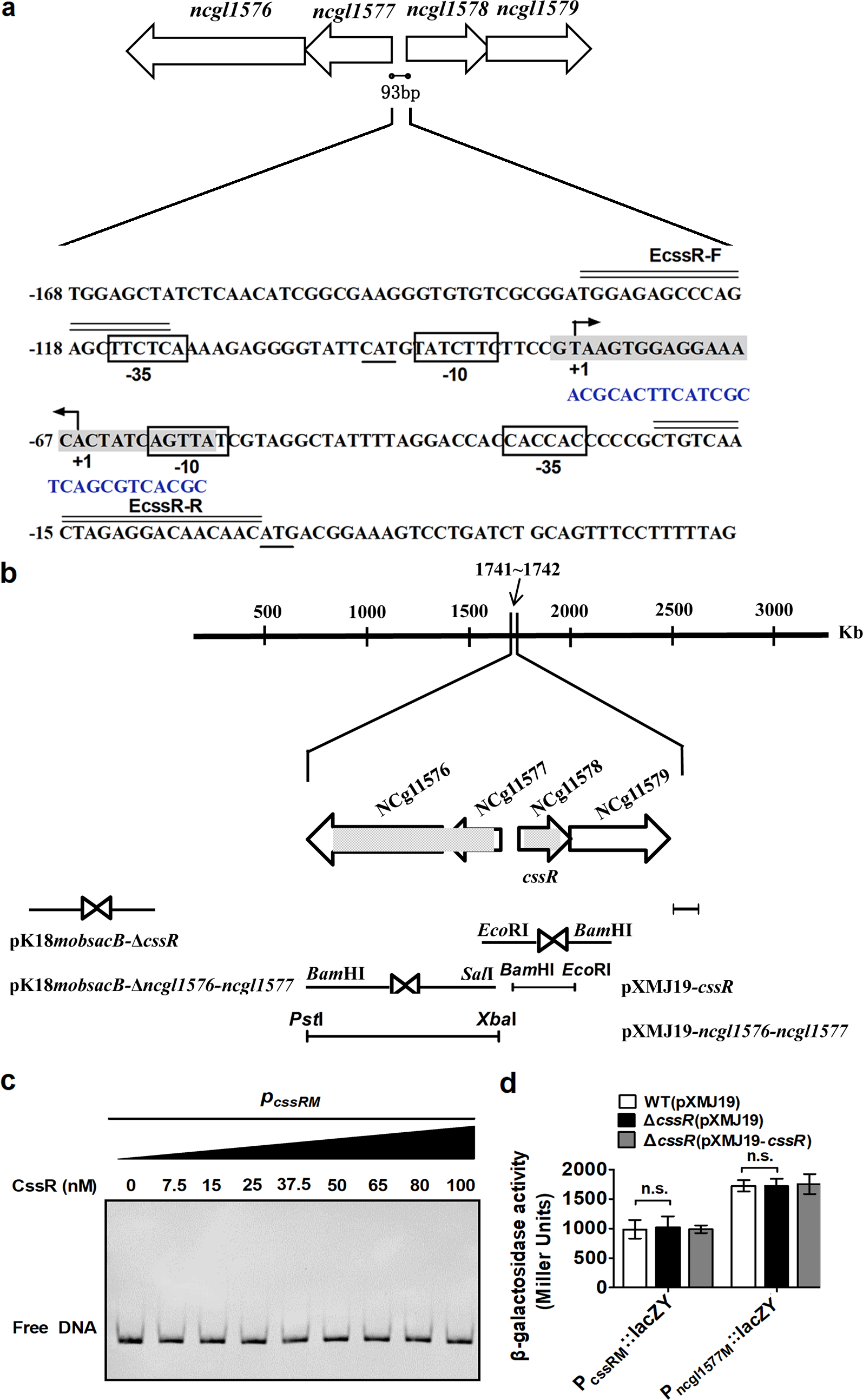


**Figure S2 Detailed genetic maps of the regulatory region of CssR. (a)** The intergenic region was the upstream of *cssR* and *ncgl1577* genes. Shaded gray indicated the CssR binding site identified based on the DNase I footprinting assay. The sequence used to replace the binding site was shown in blue below the promoter sequence. +1 denoted the putative transcription start point. The deduced -35 and -10 promoter elements were boxed. The start codons of *cssR* and *ncgl1577* were indicated with underscore. A 131-bp EMSA DNA promoter fragment (131-bp *P_cssR_*) was obtained using primers EcssR-F and EcssR-R. The sequence of primers was double underlined. **(b)** Physical map of the *cssR*(*ncgl1578*)-*ncgl1579* and *ncgl1576-ncgl1577* operons in *Corynebacterium glutamicum* RES167 parental strain (WT) and construction of plasmids for gene disruption (pK18*mobsacB* derivatives) or complementation (pXMJ19 derivatives). Open reading frames (ORFs) were marked by open arrows, and the deleted regions were in grey. The restriction sites were indicated. (**c**)The interaction between His_6_-CssR and the 131-bp promoter mutating the identified CssR binding region (131-bp *P_cssRM_*). (**d**) Mutation in the identified CssR binding site derepressed the *cssR* and *ncgl1577* expression. Relative levels of transcripts were presented as the mean values ± SD calculated from three sets of independent experiments. n.s., no significance.


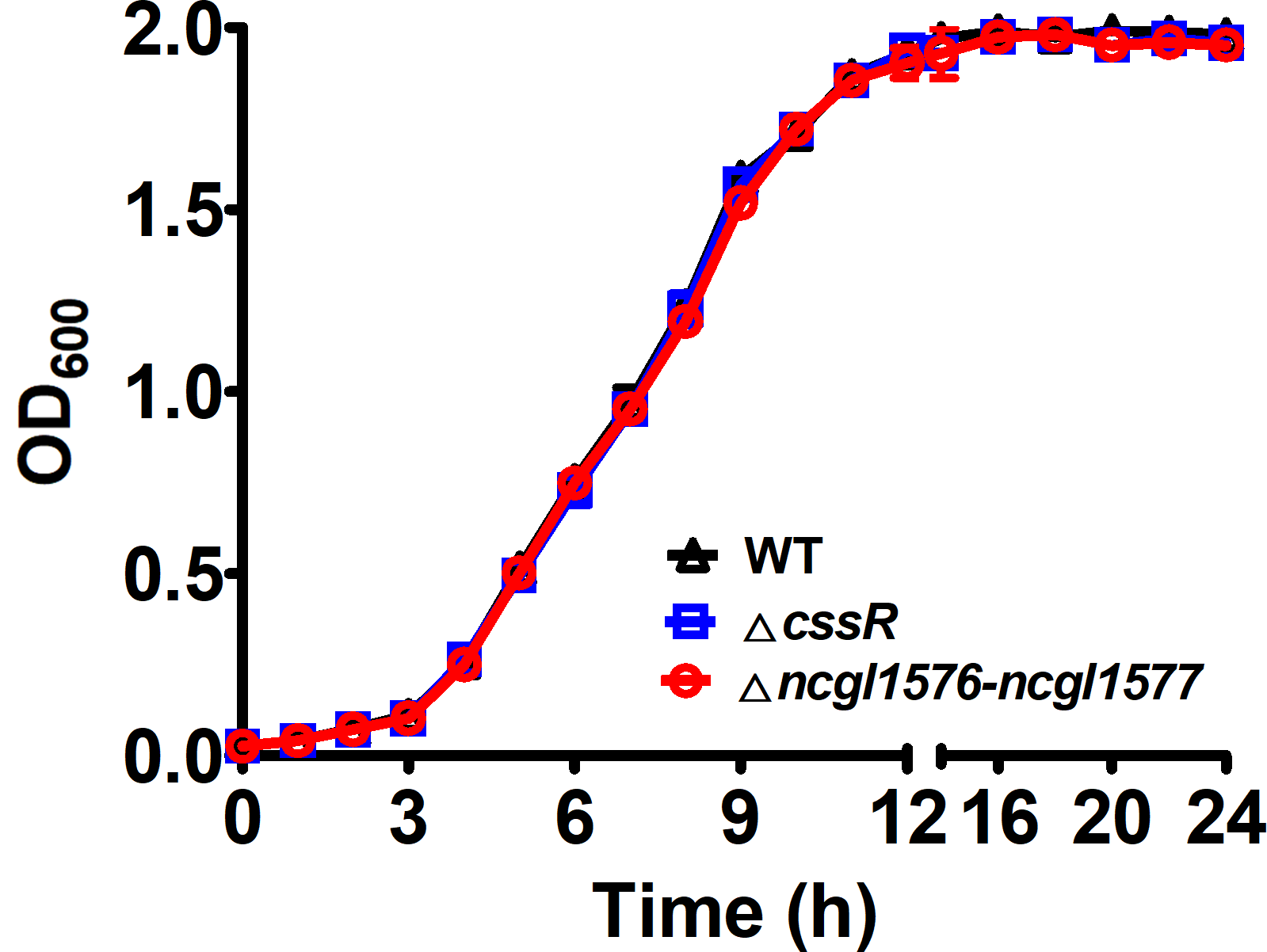


**Figure S3 Growth curves of the WT strain (the *C. glutamicum* RES167 parental strain), Δ*cssR* mutant (the mutant lacking *cssR*), and Δ*ncgl1576-ncgl1577* mutant (the mutant lacking *ncgl1576-ncgl1577*) under normal conditions.** The growth of the indicated strains in LB media was monitored by measuring OD_600_ at indicated time points.


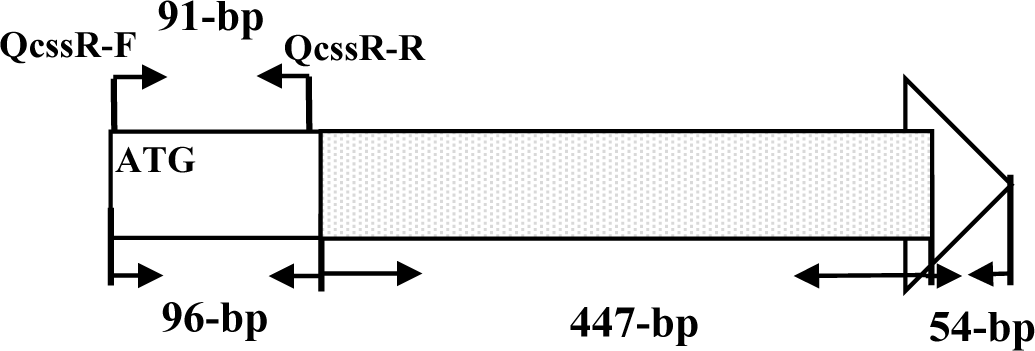


**Figure S4** 91-bp *cssR* transcript (corresponding to nucleotides +1 to +91 relative to the translational start codon (ATG) of *cssR* gene) was amplified from the remaining *cssR* ORF in Δ*cssR* mutant with primers Q_cssR_-F and Q_cssR_-R. *cssR* ORF was marked by open arrow and the deleted region was in grey.


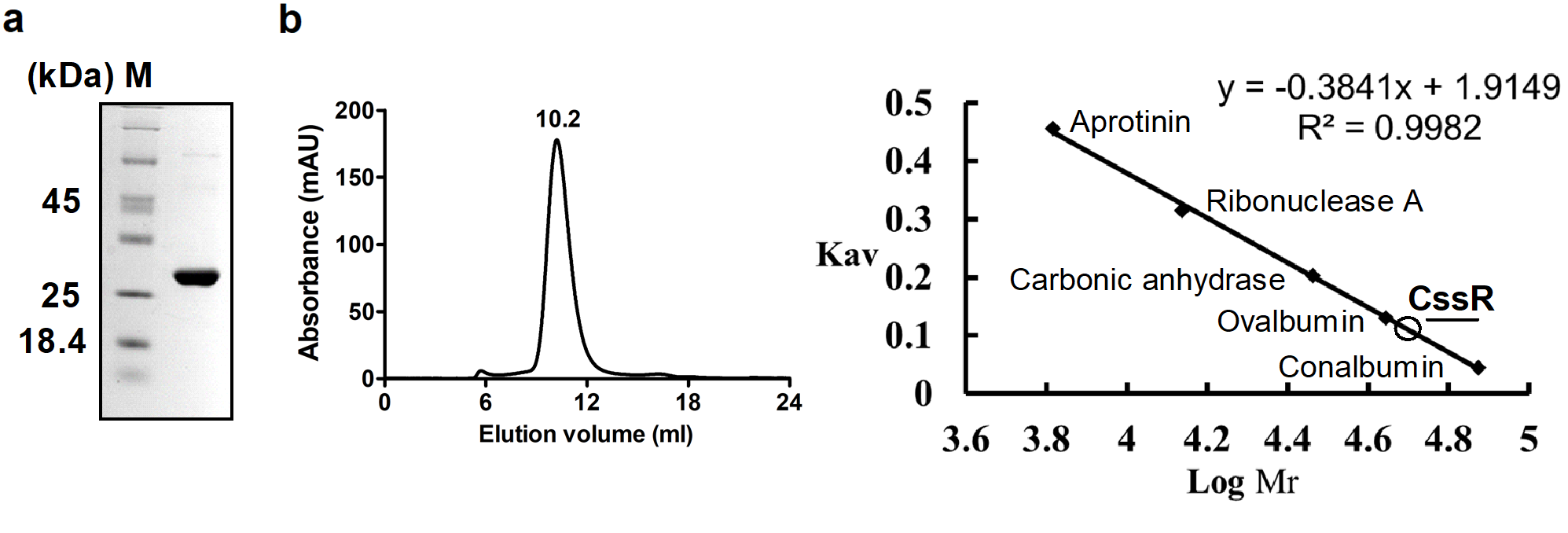


**Figure S5 Purification of His_6_-CssR and determination of the native molecular mass by size exclusion chromatography. (a)** Coomassie-stained SDS-PAGE of His_6_-CssR purified by Ni^2+^-NTA chromatography. **(b)** Native CssR protein was separated on a Superdex 75 10/300 GL column. Elution of native CssR from size exclusion column (left panel). For calibration, a premixed protein molecular mass marker containing the following proteins was used: aprotinin (6,500 Da), ribonuclease A (13,700 Da), carbonic anhydrase (29,000 Da), ovalbumin (44,000 Da), and conalbumin (75,000 Da). *V_o_* was determined with blue dextran (2,000 kDa). Hollow circle represented the position of CssR (right panel)


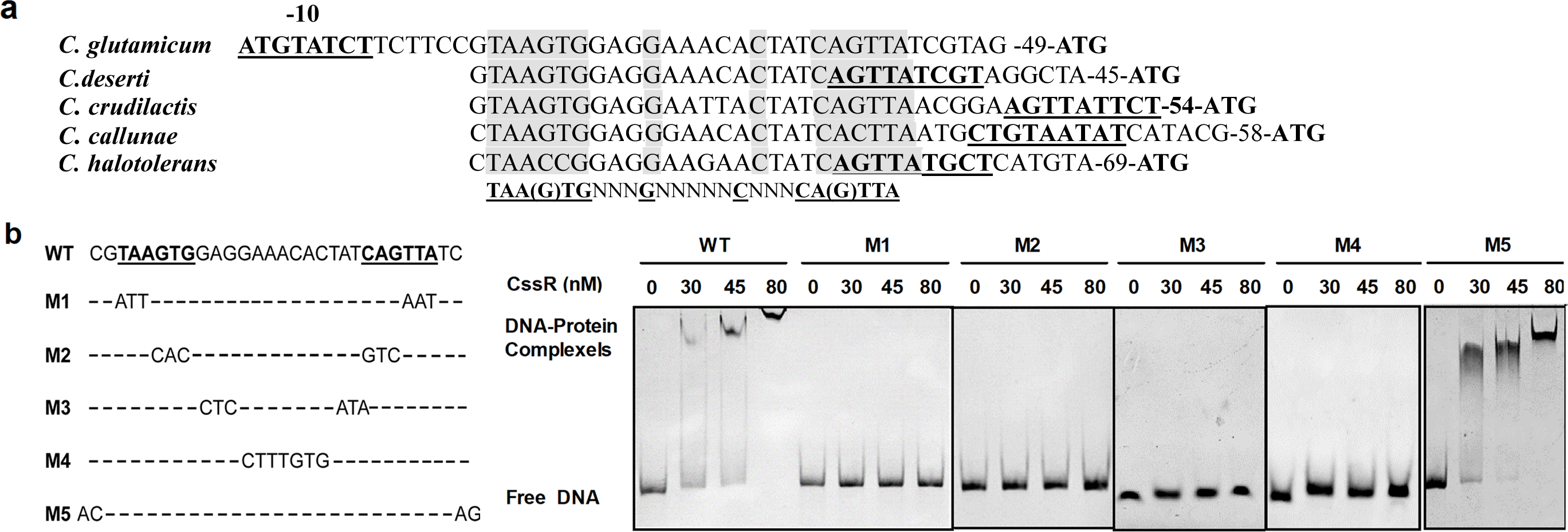


**Figure S6 Sequence of the *cssR* promoter region of *C. glutamicum* aligned to putative *cssR* promoter regions from other *Corynebacterium* species.** (**a**) Indicated were the start of the *cssR* coding region (start codons bold), putative -10 (underlined and bold), and the CssR binding site as determined by DNase I footprints (shaded in gray) in the intergenic region between *cssR* and *ncgl1577* genes. As shown by the alignment, also the other species possessed putative CssR binding sites in the intergenic region between *cssR* and *ncgl1577* genes. The binding site represented an imperfect inverted repeat with the consensus sequence TAA(G)TGN_3_GN_5_CN_3_CA(G)TTA**.** (**b**) The 29-bp sequence shown beside these fragments included the proposed CssR consensus binding site (bold and underlined) and four base pairs upstream and downstream. CssR binding site was analyzed by mutational analysis. The mutations M1-M5 were introduced by PCR and were shown below the wild-type (WT) sequence. The corresponding DNA fragments were analyzed by EMSAs with CssR.


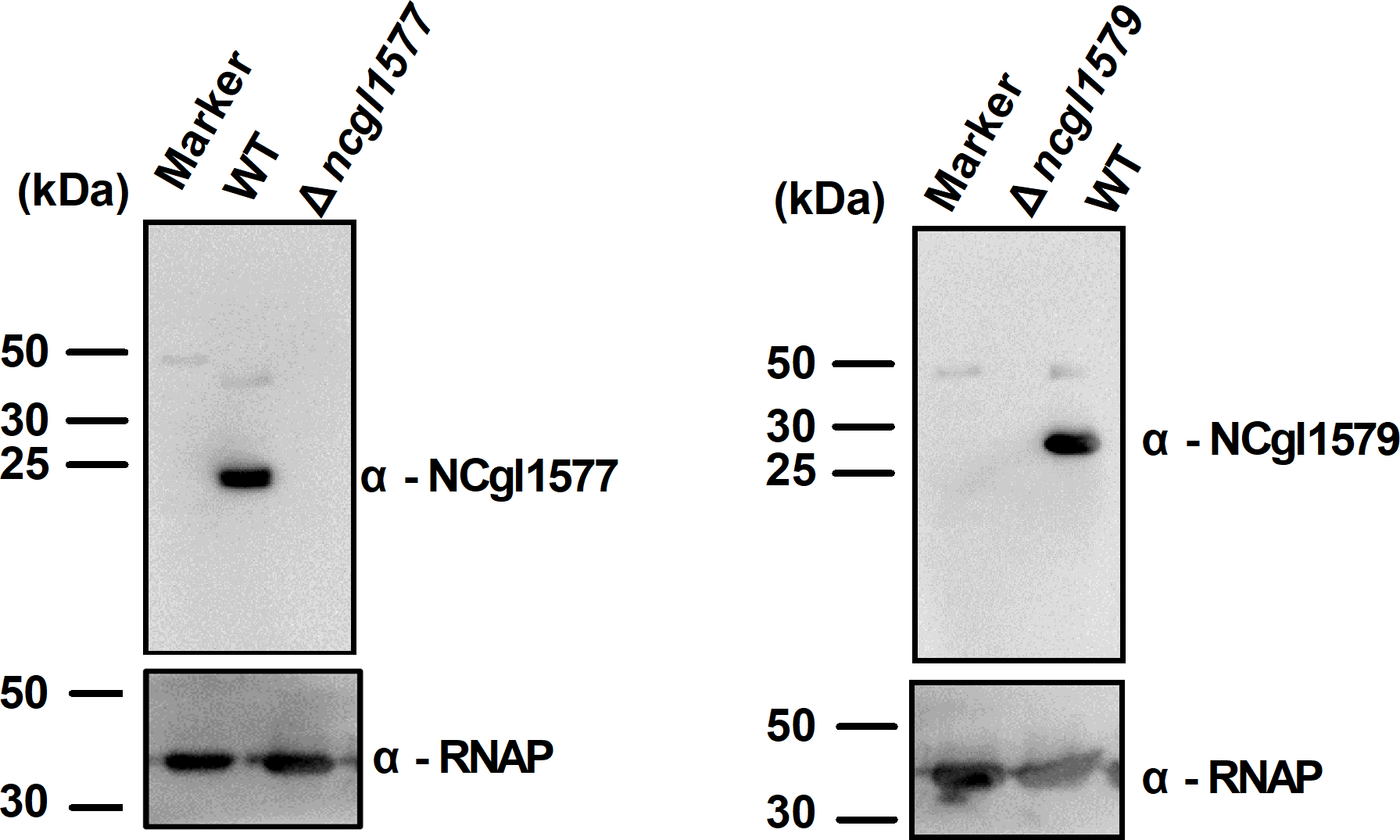


**Figure S7 The NCgl1577 and NCgl1579 were examined in *C. glutamicum*.** Anti-NCgl1577 and -NCgl1579 antibodies detected a single protein with a mobility consistent with the predicted size that was absent from Δ*ncgl1577* and Δ*ncgl1579* mutants. Antibody to anti-cytosolic RNA polymerase α (α-RNAP) was used as a loading control.


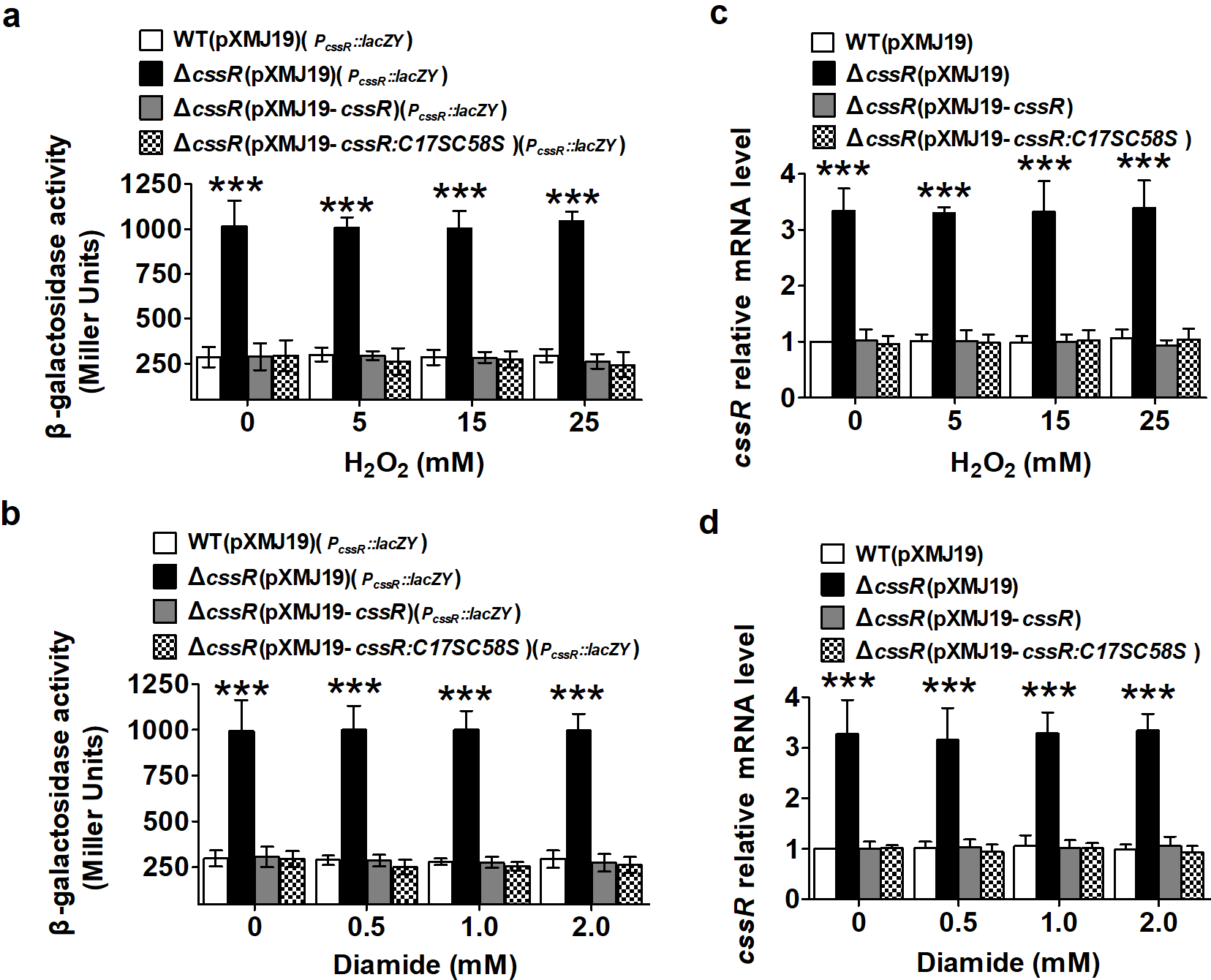


**Figure S8** **Negative regulation of *cssR* by CssR.** **(a and b)** β-galactosidase analyses of the *cssR* promoter (*P_cssR_*) activities by using the transcriptional *P_cssR_::lacZY* chromosomal fusion reporter expressed in WT(pXMJ19), Δ*cssR*(pXMJ19) mutant, and complementary Δ*cssR*(pXMJ19-*cssR*) under H_2_O_2_ and diamide conditions. **(c and d)** Quantitative RT-PCR analyses of *cssR* expression in WT(pXMJ19), Δ*cssR*(pXMJ19) mutant, and complementary Δ*cssR*(pXMJ19-*cssR*) strains under indicated conditions. Results were the average of three independent experiments; the standard deviation was indicated by bars. The mRNA levels were presented relative to the value obtained from WT(pXMJ19) cells without stress treatment. Relative transcript levels of WT(pXMJ19) strains without stress treatment were set at a value of 1.0. Data shown were the averages of three independent experiments, and error bars indicated the SDs from three independent experiments. ***, *P* < 0.001.


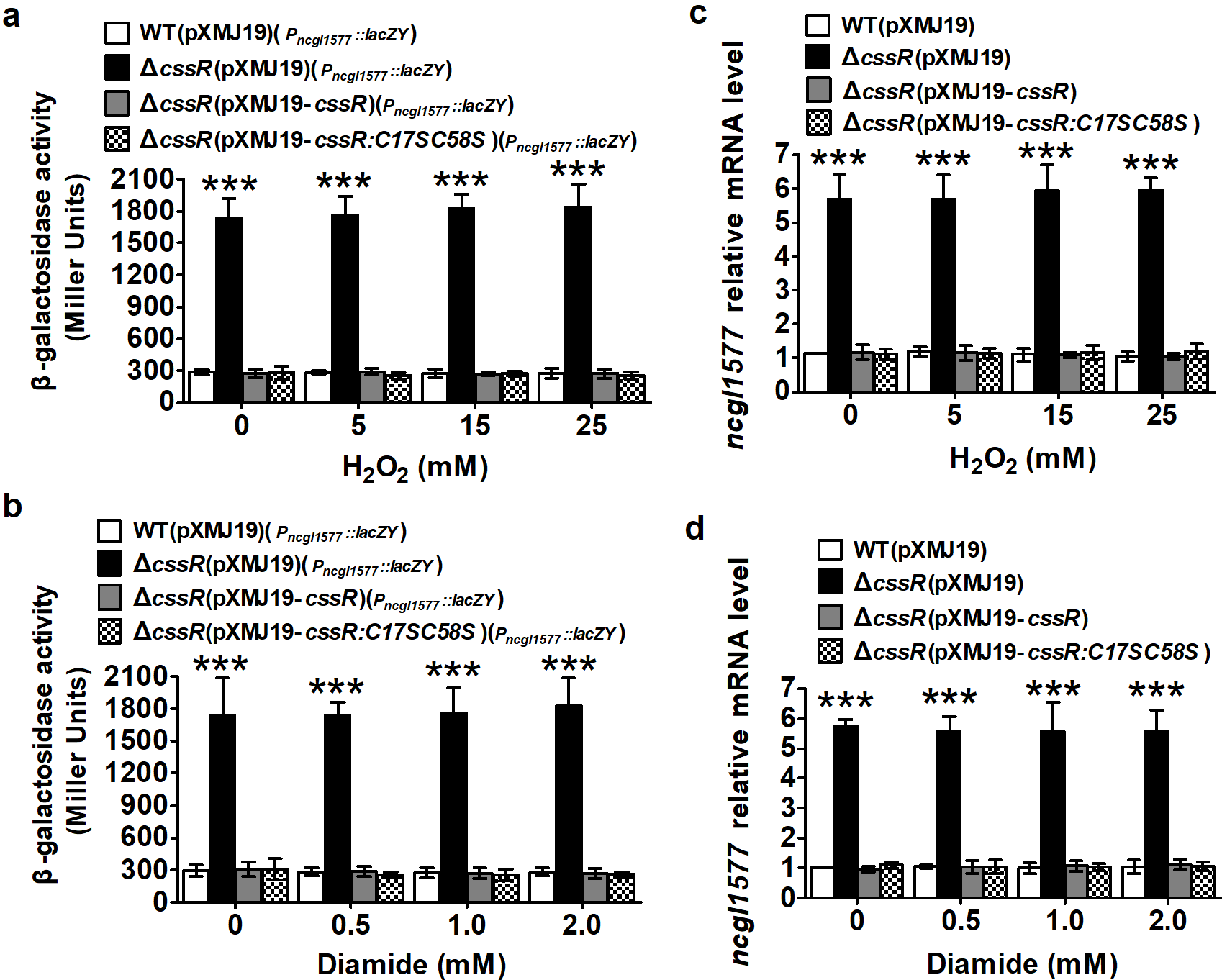


**Figure S9** **Negative regulation of *ncgl1577* by CssR.**  **(a and b)** β-galactosidase analyses of the *ncgl1577* promoter (*P_ncgl1577_*) activities by using the transcriptional *P_ncgl1577_::lacZY* chromosomal fusion reporter expressed in WT(pXMJ19), Δ*csnR*(pXMJ19) mutant, and complementary Δ*cssR*(pXMJ19-*cssR*) under H_2_O_2_ and diamide conditions. **(c and d)** Quantitative RT-PCR analyses of *ncgl1577* expression in WT(pXMJ19), Δ*cssR*(pXMJ19) mutant, and complementary Δ*cssR*(pXMJ19-*cssR*) strains under H_2_O_2_ and diamide conditions. Results were the average of three independent experiments; the standard deviation was indicated by bars. The mRNA levels were presented relative to the value obtained from WT(pXMJ19) cells without stress treatment. Relative transcript levels of WT(pXMJ19) strains without stress treatment were set at a value of 1.0. Data shown were the averages of three independent experiments, and error bars indicated the SDs from three independent experiments. ***, *P* < 0.001.


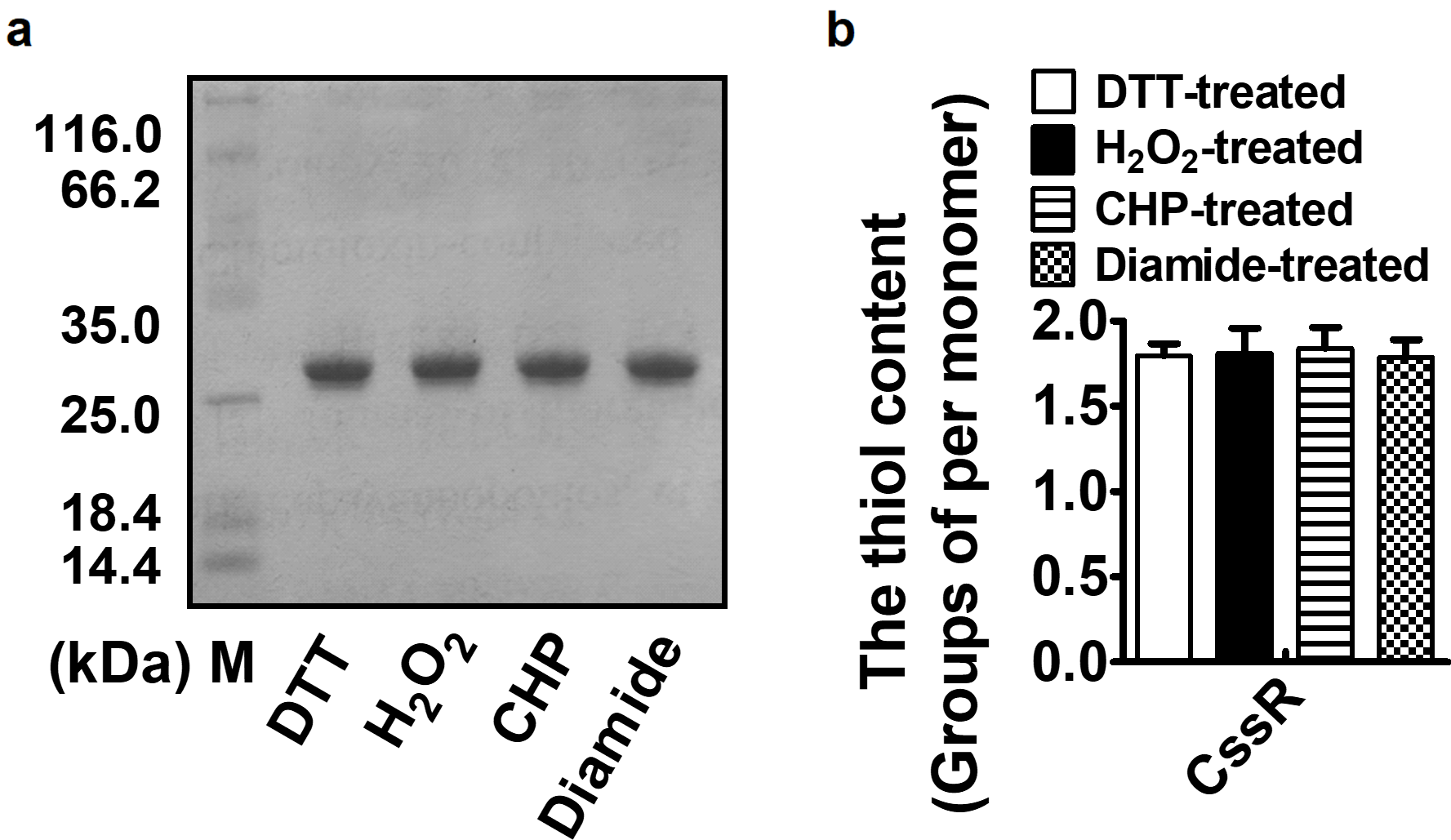


**Figure S10 Redox response of CssR *in vitro*. (a)** Redox response of CssR detected by nonreducing SDS-PAGE. 20 μM proteins treated with 50 mM DTT were further incubated with or without 100 mM H_2_O_2_, 80 μM CHP, and 60 μM diamide for 30 min, respectively, and samples were then separated by 15% nonreducing SDS-PAGE and stained with Coomassie Brilliant Blue. M represented protein molecular mass marker. **(b)** Quantification of free CssR thiol levels. DTT-, H_2_O_2_-, CHP-, and diamide-treated CssR (10 μM) were mixed 2 mM with DTNB in 50 mM Tris-HCl buffer (pH 8.0), respectively, and the absorbance was monitored at 412 nm against a 2 mM DTNB solution as reference. These data were means of the values obtained from three independent assays.
